# Time‐Resolved SAXS Studies During the Synthesis of Hydrolytically Degradable Poly(ε‐caprolactone)‐Poly(*N,N′*‐dimethylacrylamide) Diblock Copolymer Nanoparticles in Aqueous Media

**DOI:** 10.1002/anie.202524000

**Published:** 2025-11-17

**Authors:** Matthew A. H. Farmer, Oleksandr O. Mykhaylyk, Olga Shebanova, Osama M. Musa, Steven P. Armes

**Affiliations:** ^1^ Dainton Building, School of Mathematical and Physical Sciences The University of Sheffield Brook Hill Sheffield South Yorkshire UK; ^2^ Diamond Light Source Ltd, Diamond House, Harwell Science and Innovation Campus Didcot Oxfordshire OX11 0DE UK; ^3^ Ashland Specialty Ingredients 1005 US 202/206 Bridgewater New Jersey 08807 USA

**Keywords:** Aqueous dispersions, Block copolymer nanoparticles, RAFT polymerization, *Reverse sequence* polymerization‐induced self‐assembly, Time‐resolved small‐angle X‐ray scattering

## Abstract

The mechanism of nanoparticle formation during *reverse sequence* polymerization‐induced self‐assembly (PISA) is studied by small‐angle X‐ray scattering (SAXS). More specifically, *N,N′*‐dimethylacrylamide (DMAC) monomer is added to a trithiocarbonate‐capped poly(ɛ‐caprolactone) (PCL) precursor and initially polymerized in the bulk at 80 °C via reversible addition‐fragmentation chain transfer (RAFT) polymerization. SAXS indicates the unexpected formation of molten PCL droplets dispersed within DMAC monomer. After 5 min (14% DMAC conversion) at 80 °C, the reaction mixture is diluted with water, and the aqueous milieu is analyzed using a flow cell. A transient lamellar phase is formed immediately after water addition that subsequently transforms into nascent spherical nanoparticles. During the remaining DMAC polymerization, the overall nanoparticle diameter remains essentially constant with a concomitant reduction in the PCL core radius and the aggregation number. This suggests that individual PCL‐PDMAC chains are in equilibrium with the nanoparticles. SAXS analysis indicates that the amorphous PCL cores have a mean diameter of 8.8 nm at 80 °C: X‐ray diffraction (XRD) studies confirm that such nanoscale confinement prevents their crystallization on cooling to 20 °C. Finally, this formulation can be combined with crystallization‐driven self‐assembly (CDSA): UV‐initiated DMAC polymerization at 15 °C produces rod‐like PCL‐PDMAC nanoparticles with semicrystalline PCL cores.

## Graphical abstract

Synthesis of hydrolytically degradable poly(*N,N′*‐dimethylacrylamide)‐poly(ɛ‐caprolactone) nanoparticles via *reverse sequence* polymerization‐induced self‐assembly (PISA) at 80 °C is studied by time‐resolved small‐angle X‐ray scattering (SAXS). This technique reveals the formation of molten poly(ɛ‐caprolactone) (PCL) droplets prior to water addition, a transient lamellar phase, and the mechanism of growth for the final spherical block copolymer nanoparticles.

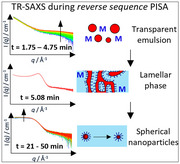

## Introduction

Poly(ε‐caprolactone) (PCL) is an FDA‐approved polymer that is widely recognized to be hydrolytically degradable and biocompatible.^[^ ^1^ ^]^ Accordingly, PCL‐based nanoparticles have been evaluated in cosmetics,^[^ ^2^, ^3^, ^4^, ^5^ ^]^ for drug delivery systems^[^ ^6^, ^7^, ^8^, ^9^, ^10^ ^]^ and as injectable hydrogels.^[^ ^11^, ^12^, ^13^, ^14^, ^15^, ^16^ ^]^ PCL‐based diblock copolymers are typically synthesized by ring‐opening polymerization (ROP) of ε‐caprolactone using a hydroxy‐capped poly(ethylene oxide) (PEO) precursor either in the bulk or in an aprotic solvent such as chloroform.^[^ ^17^, ^18^, ^19^, ^20^ ^]^ Subsequent nanoprecipitation or solvent evaporation is then used to produce spherical PCL‐core nanoparticles in aqueous media.^[^ ^8^, ^17^ ^]^ However, such post‐polymerization processing methods usually only produce dilute dispersions of PCL nanoparticles, which is not conducive to industrial scale‐up. Clearly, a more process‐intensive route to generate concentrated aqueous dispersions of hydrolytically degradable PCL‐based nanoparticles would constitute an important advance. Furthermore, the formation of anisotropic PCL‐based nanoparticles (e.g., rods) should be beneficial because such morphologies are likely to confer advantages as Pickering emulsifiers.^[^ ^21^, ^22^, ^23^, ^24^ ^]^ In this context, it is noteworthy that ellipsoidal polystyrene latexes with higher aspect ratios are more effective stabilizers than the equivalent spherical latex particles for both oil‐in‐water and water‐in‐oil emulsions.^[^ ^25^ ^]^

It is well known that block copolymers comprising a semicrystalline block can undergo crystallization‐driven self‐assembly (CDSA) to form highly anisotropic nanoparticles such as rods or platelets.^[^ ^26^, ^27^, ^28^, ^29^, ^30^, ^31^, ^32^ ^]^ Initially, CDSA was based on rather esoteric, non‐degradable semicrystalline polymers such as poly(ferrocenyldimethylsilane).^[^ ^26^, ^27^, ^28^, ^29^ ^]^ However, PCL,^[^ ^33^, ^34^, ^35^, ^36^, ^37^ ^]^ poly(*L*‐lactide) (PLLA)^[^ ^30^, ^31^, ^32^ ^]^ or poly(δ‐valerolactone) (PVL)^[^ ^37^ ^]^ can also be used for CDSA. Notably, the mean nanoparticle aspect ratio can be precisely controlled.^[^ ^31^, ^35^ ^]^

For example, the length/width ratio of poly(ε‐caprolactone)‐poly(*N,N′*‐dimethylacrylamide) (PCL‐PDMAC) rods can be adjusted by systematically varying the number of PCL‐PDMAC chains (initially molecularly dissolved in THF) that are added to an alcoholic dispersion of “seed” PCL‐PDMAC nanoparticles.^[^ ^35^ ^]^ More specifically, the mean rod length is proportional to the concentration of soluble copolymer chains.^[^ ^35^ ^]^ Furthermore, when poly(methyl methacrylate) was incorporated into the copolymer as a third (amorphous) block, PCL‐PMMA‐PDMAC rods of adjustable aspect ratio could be obtained directly in aqueous media.^[^ ^35^ ^]^ Inspecting the literature, the preferred morphology for PCL‐PDMAC nanoparticles appears to be rods.^[^ ^34^, ^35^ ^]^ However, 2D platelets can be formed by adding PCL homopolymer during the CDSA processing step, although only rather dilute copolymer dispersions are typically obtained.^[^ ^33^, ^37^ ^]^ On the other hand, CDSA has been recently used to prepare concentrated dispersions of highly anisotropic nanoparticles in either aqueous media^[^ ^38^ ^]^ or organic solvents such as dichloromethane or toluene.^[^ ^39^, ^40^ ^]^

Recently, we reported a so‐called *reverse sequence* polymerization‐induced self‐assembly (PISA) formulation, whereby aqueous dispersions of PCL‐PDMAC spherical nanoparticles can be prepared at up to 25% w/w solids.^[^ ^41^ ^]^ In a traditional PISA synthesis conducted in aqueous media,^[^ ^42^, ^43^, ^44^ ^]^ a water‐soluble precursor is chain‐extended with a suitable monomer that forms a water‐insoluble polymer at some critical degree of polymerization (DP).^[^ ^45^, ^46^ ^]^ This leads to in situ self‐assembly and the formation of concentrated dispersions of spheres, worms, or vesicles.^[^ ^47^, ^48^, ^49^, ^50^, ^51^, ^52^, ^53^ ^]^ However, for a *reverse sequence* aqueous PISA synthesis, the hydrophobic block is prepared first and is either (i) stabilized in the form of latex particles in aqueous media by employing an ionic reversible addition‐fragmentation chain transfer (RAFT) agent to confer surface charge^[^ ^54^, ^55^, ^56^ ^]^ or (ii) solubilized in highly concentrated aqueous media using a suitable hydrophilic monomer as a cosolvent.^[^ ^41^, ^57^, ^58^ ^]^ For example, we recently reported that a trithiocarbonate‐capped PCL precursor can be dissolved in minimal DMAC monomer to produce an 80% w/w aqueous solution at 80 °C.^[^ ^41^ ^]^ Subsequently, RAFT polymerization^[^ ^59^ ^]^ of DMAC produced amphiphilic PCL‐PDMAC diblock copolymer chains. At an appropriate *intermediate* DMAC monomer conversion, preheated degassed water was added to the reaction mixture to induce self‐assembly to form nascent nanoparticles, and the DMAC polymerization was allowed to continue to completion. However, only spherical nanoparticles could be obtained under such conditions.^[^ ^41^ ^]^ This is because the synthesis temperature (80 °C) exceeds the melting temperature of the crystalline domains, *T*_m_, for the PCL chains (45 °C; see Figure S1), so in situ CDSA cannot occur.

**Figure 1:**
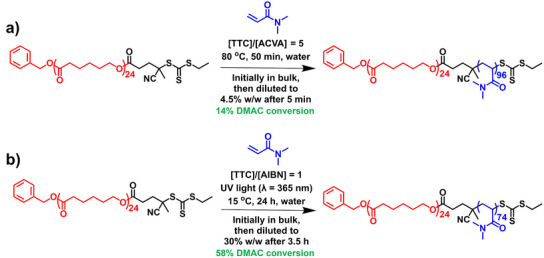
Summary of the two nanoparticle formulations reported in this study. a) Synthesis of PCL_24_‐PDMAC_96_ nanoparticles prepared at 80 °C via *reverse sequence* aqueous PISA. Initially, the RAFT polymerization of DMAC is conducted in the bulk, with subsequent dilution to 4.5% w/w solids after 5 min (14% DMAC conversion) using preheated deoxygenated deionized water. Conditions: [PCL_24_‐TTC]/[ACVA] molar ratio = 5.0, target DP = 100. b) Synthesis of PCL_24_‐PDMAC_74_ nanoparticles prepared via UV‐initiated polymerization at 15 °C by combining *reverse sequence* aqueous PISA with CDSA. Initially, the RAFT polymerization of DMAC is conducted in the bulk, with subsequent dilution to 30% w/w solids using deoxygenated deionized water after 3.5 h (58% DMAC conversion). Conditions: [PCL_24_‐TTC]/[AIBN] molar ratio = 1.0, target DP = 80.

Herein we revisit the *reverse sequence* aqueous PISA synthesis of PCL‐PDMAC diblock copolymer nanoparticles using time‐resolved small‐angle X‐ray scattering (TR‐SAXS) to study the mechanism of nanoparticle formation at 80 °C, see Figure 1a. Furthermore, UV‐initiated RAFT polymerization of DMAC is used to prepare PCL‐PDMAC nanoparticles at 15 °C when targeting up to 30% w/w solids; see Figure 1b. In this case, the reaction temperature is well below the *T*_m_ for the PCL chains, so CDSA can influence the final nanoparticle morphology.

## Results and Discussion

### Time‐Resolved SAXS Studies of the Reverse Sequence PISA Synthesis of PCL_24_‐PDMAC_96_ Spherical Nanoparticles at 80 °C

Monohydroxy‐capped poly(ε‐caprolactone) (hereafter denoted PCL_24_‐OH) was prepared via anionic ROP of ε‐caprolactone using benzyl alcohol as an initiator. This homopolymer was subsequently esterified using a carboxylic acid‐functionalized trithiocarbonate‐based (TTC) RAFT agent.^[^ ^41^ ^]^

^1^H NMR spectroscopy and UV GPC analysis were used to (i) calculate the mean DP of the resulting PCL_24_‐TTC precursor, (ii) determine its degree of esterification (∼100%), (iii) assess its molecular weight distribution (*M*_w_/*M*_n_ = 1.15), and (iv) confirm that no residual unreacted RAFT agent remained after purification (see Figures S2–S4).

**Figure 2:**
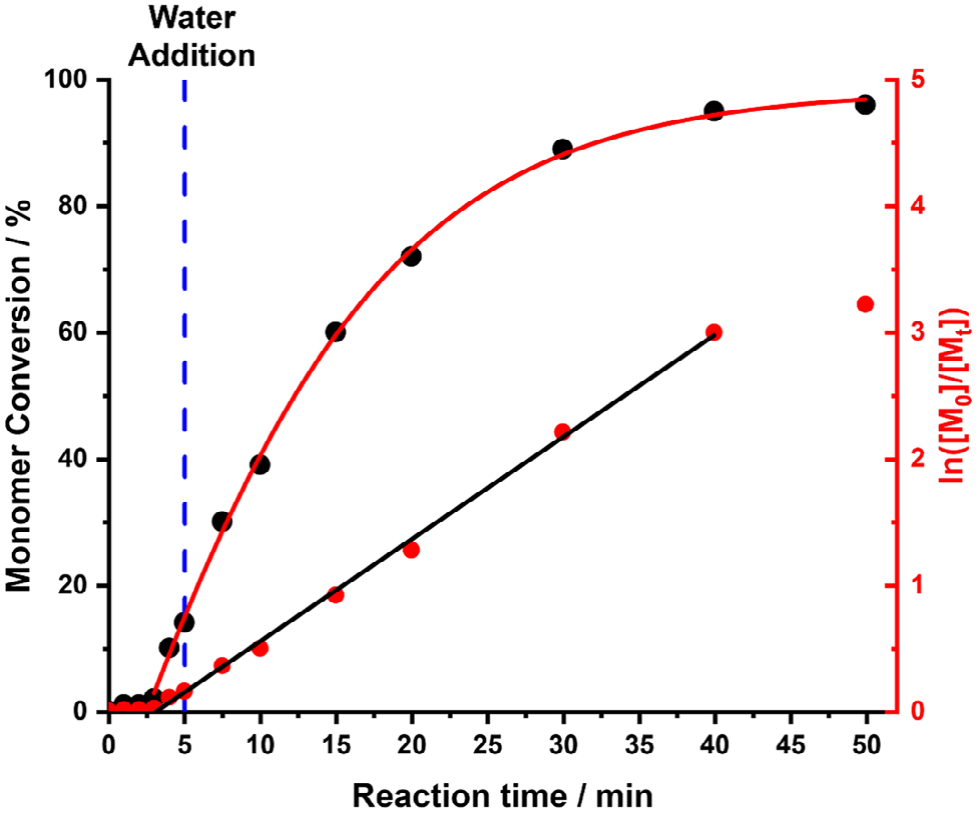
Kinetics of polymerization obtained by ^1^H NMR spectroscopy (black points) and the corresponding semi‐logarithmic plot (red points) for the laboratory‐scale synthesis of PCL_24_‐PDMAC_100_ nanoparticles, whereby DMAC polymerization was initiated in the bulk at 80 °C followed by dilution with preheated degassed deionized water after precisely 5 min (DMAC conversion = 14%) to target 4.5% w/w solids. Conditions: [PCL_24_‐TTC]/[ACVA] molar ratio = 5.0.

For time‐resolved SAXS measurements, the initial reaction mixture comprising the PCL_24_‐TTC precursor, azo initiator, and DMAC monomer was loaded into a glass vial. This hydrophobic PCL precursor was chain‐extended via DMAC polymerization at 80 °C, which was initially conducted in the bulk. Subsequently, this reaction mixture was diluted to 4.5% w/w solids via rapid addition of preheated degassed deionized water. Optimization experiments established that water addition was required after precisely 5 min for this formulation (see later). ^1^H NMR kinetic data were also obtained for the same *reverse sequence* aqueous PISA synthesis performed on a larger scale to allow periodic sampling. The intermediate DMAC conversion was determined to be approximately 14% at the point of water addition (see Figure 2), which corresponds to an instantaneous diblock copolymer composition of PCL_24_‐PDMAC_14_.

Notably, the approximate twenty‐fold dilution of the polymerizing milieu with water does not result in a slower polymerization. Indeed, the corresponding semilogarithmic plot indicates that the DMAC polymerization proceeds with first‐order kinetics at essentially the same rate before and after dilution with water. This is because the rate of polymerization of DMAC is significantly faster in aqueous solution compared to that in the bulk.^[^ ^60^ ^]^

**Figure 3:**
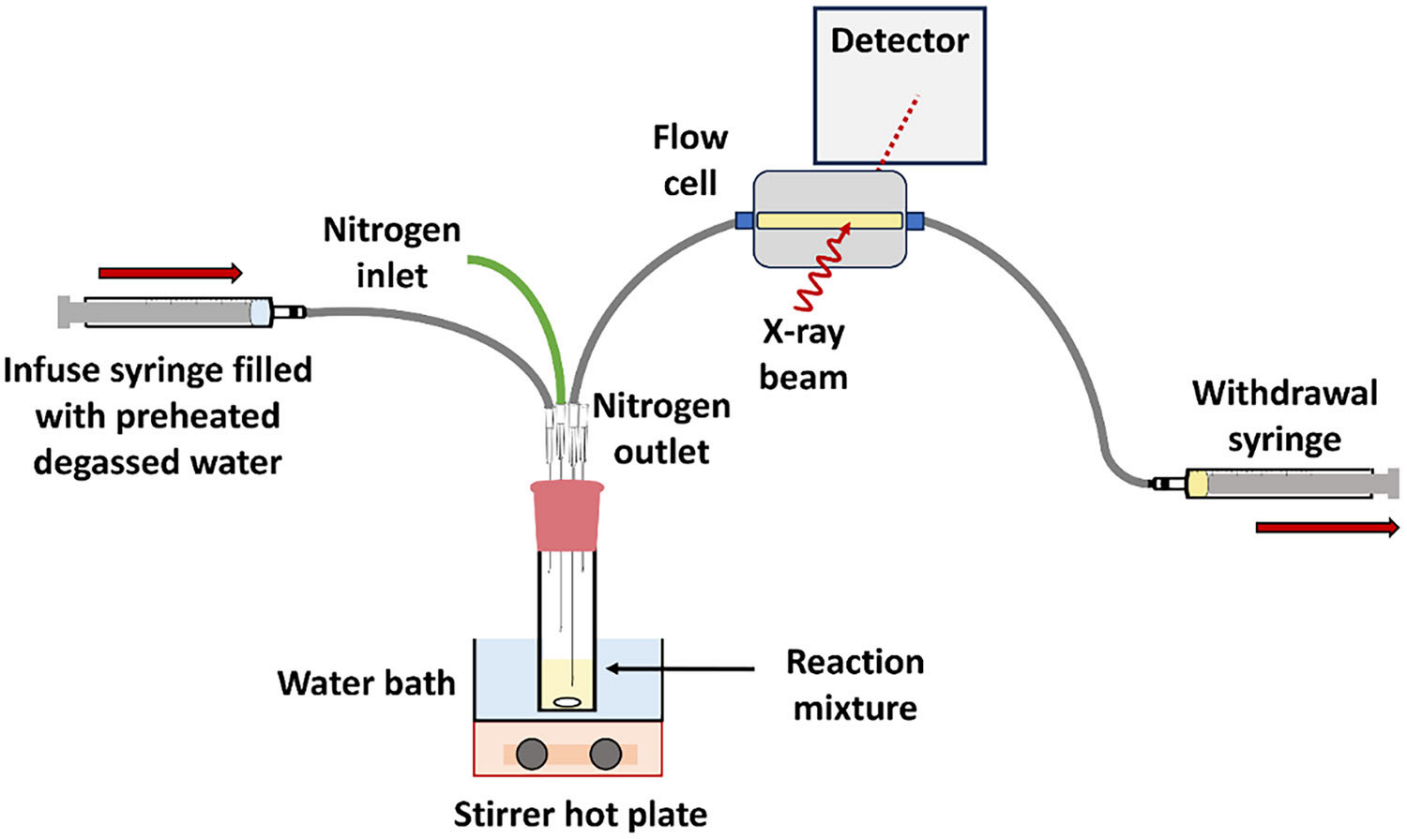
Schematic representation of the experimental setup used to record time‐resolved SAXS patterns during the RAFT polymerization of DMAC, initially in the bulk at 80 °C followed by dilution to 4.5% w/w solids after 5 min using preheated degassed deionized water. Conditions: [PCL_24_‐TTC]/[ACVA] molar ratio = 5.0, PDMAC target DP = 100.

Owing to health and safety constraints when performing synchrotron SAXS experiments, dilution of the reaction mixture via addition of water was performed using a remote‐controlled syringe pump (see Figure 3). Moreover, a second syringe pump was employed to continuously withdraw the polymerizing solution, which was passed through an SAXS capillary flow cell for in situ analysis.^[^ ^61^ ^]^ This experimental setup ensured that the reaction vessel was not continuously exposed to X‐rays, which eliminates the possibility of rate acceleration owing to an additional radical flux generated by the intense synchrotron beam.^[^ ^62^ ^]^

To enable continuous sampling of the reaction mixture, both the time at which water was added and the final copolymer concentration had to be optimized to ensure that the tubing that connected the reaction mixture to the flow cell did not become blocked as the reaction mixture became more viscous. For example, adding water after 4.0 min resulted in macroscopic precipitation of the PCL precursor because the growing PDMAC chains were too short to confer effective steric stabilization on the nascent PCL‐PDMAC nanoparticles. However, if water was added after 6.0 min, the reaction mixture became too viscous and blocked the tubing, which prevented sampling of the reaction mixture. Empirically, it was found that diluting the polymerizing reaction mixture to 4.5% w/w solids reduced the likelihood of tube blocking while also minimizing the structure peak within the evolving SAXS patterns (see Experimental Section for further details).

**Figure 4:**
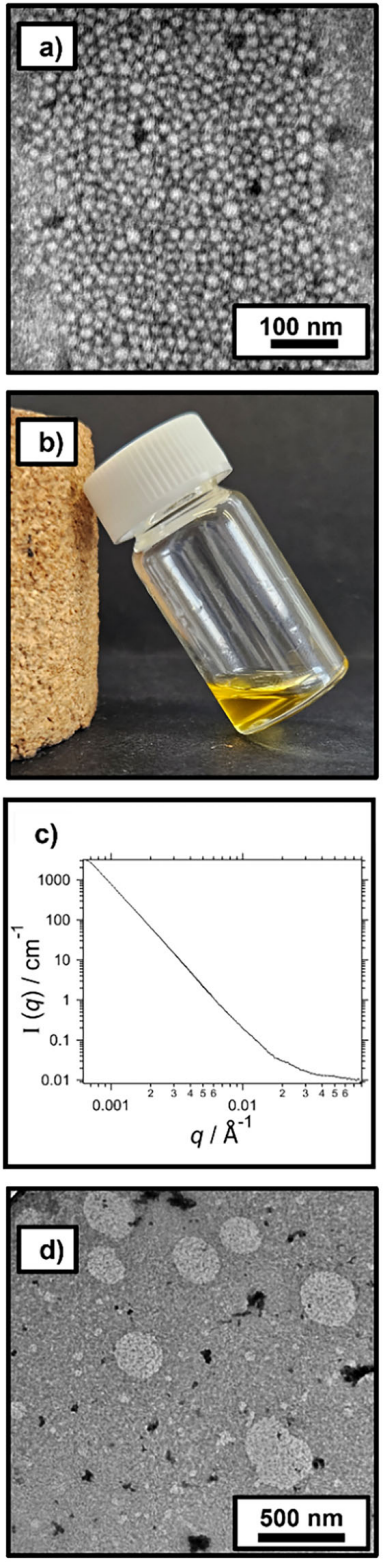
a) TEM image recorded for a 0.1% w/w aqueous dispersion of PCL_24_‐PDMAC_96_ nanoparticles synthesized via *reverse sequence* aqueous PISA at 80 °C after the TR‐SAXS study. b) Digital photograph recorded for PCL_24_‐TTC molten droplets dispersed within DMAC monomer at 80 °C. This emulsion is optically transparent owing to the very small difference in refractive index between the PCL_24_‐TTC precursor and the DMAC monomer. c) SAXS pattern recorded at 80 °C for a binary mixture of 25% w/w PCL_24_ in DMAC monomer prepared in the absence of any azo initiator (i.e., at zero DMAC conversion). d) TEM image recorded for a dilute emulsion of PCL_24_‐TTC droplets within DMAC monomer (TEM grid prepared at 80 °C).

A ^1^H NMR spectrum recorded for the final reaction mixture indicated a DMAC conversion of 96% after 50 min; see Figure S5. Furthermore, GPC analysis confirmed the efficient formation of well‐defined PCL_24_‐PDMAC_96_ diblock copolymer chains (*M*_w_/*M*_n _= 1.31; minimal contamination by the PCL_24_ precursor); see Figures S6 and S7. DLS studies of the final colloidal dispersion (after dilution to 0.1% w/w) indicated the formation of nanoparticles with a z‐average diameter of 43 nm with a polydispersity index (PDI) of 0.22 (see Figure S8). Comparable DLS data were obtained in our prior study, indicating good reproducibility for this formulation.^[^ ^41^ ^]^ Given that this scattering technique reports a hydrodynamic diameter that includes the relatively long PDMAC stabilizer chains, this value is consistent with the number‐average core diameter of 18 ± 3 nm estimated from TEM analysis (Figure 4a; *ImageJ* software was used to analyze 100 nanoparticles). In summary, the *postmortem* characterization data confirmed that the *reverse sequence* aqueous PISA synthesis conducted during the TR‐SAXS experiment was successful. So what can we learn from the 320 SAXS patterns recorded during this DMAC polymerization at 80 °C?

Because the initial reaction mixture is optically transparent (see Figure 4b), we originally reported that the PCL_24_‐TTC precursor was molecularly dissolved in DMAC.^[^ ^41^ ^]^ However, SAXS analysis clearly demonstrates that this is not the case. More specifically, the pronounced upturn observed at low *q* in the SAXS pattern recorded at 80 °C prior to polymerization (i.e., for the PCL_24_‐TTC precursor plus DMAC monomer in the absence of any initiator; see Figure 4c) suggests the presence of relatively large scattering objects within the reaction mixture. Unfortunately, the size of the scattering objects cannot be estimated from the SAXS pattern shown in Figure 4c. However, the TEM image shown in Figure 4d indicates the presence of polydisperse spheres of 200–400 nm diameter. Hence it is clear that the molten PCL_24_‐TTC precursor is *not* molecularly dissolved as originally reported^[^ ^41^ ^]^ but instead forms relatively large droplets within the DMAC monomer at 80 °C. Moreover, the fortuitous refractive index (RI) match between the PCL_24_‐TTC precursor (RI = 1.48 at 80 °C) and that of the DMAC monomer (RI = 1.47 at 80 °C) accounts for the highly transparent nature of this unexpected emulsion.^[^ ^63^ ^]^

**Figure 5:**
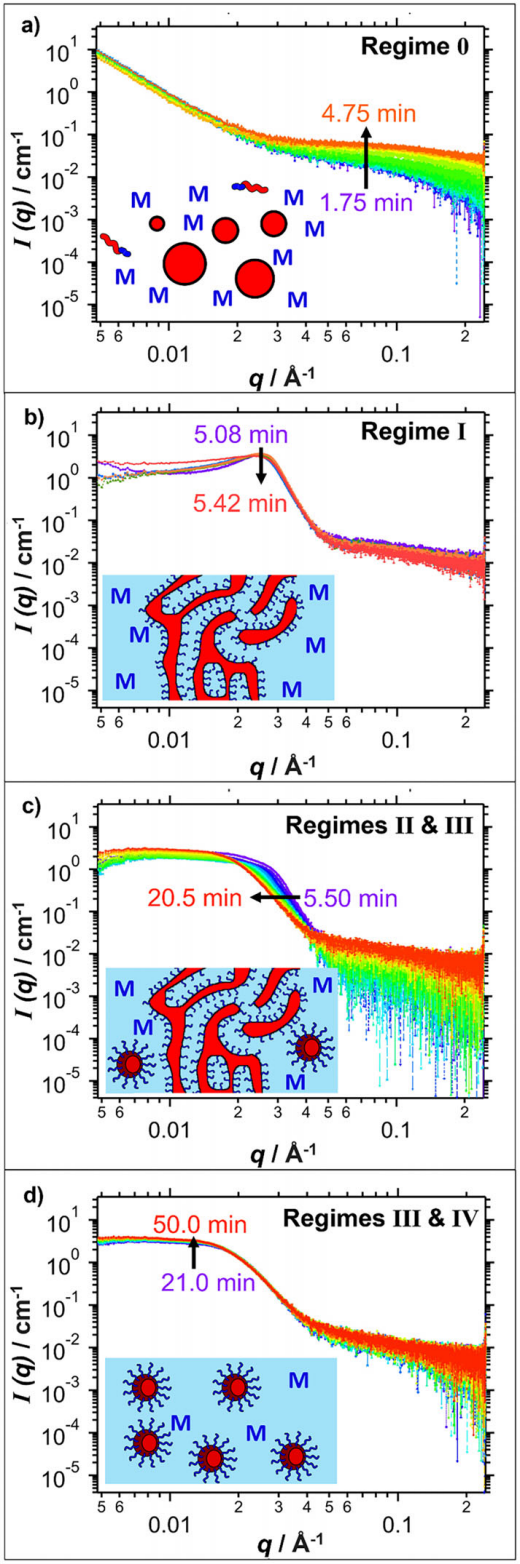
TR‐SAXS patterns recorded during the RAFT polymerization of DMAC using a PCL_24_ precursor at 80 °C (initially in the bulk, followed by dilution with water after 5.0 min) with schematic cartoons representing the scattering objects that are present in each case. This *reverse sequence* aqueous PISA formulation ultimately produced a colloidal dispersion of PCL_24_‐PDMAC_96_ spherical nanoparticles at approximately 4.5% w/w solids (96% DMAC conversion). (a) SAXS patterns recorded at 15 second intervals between 1.75 and 4.75 min. b) SAXS patterns recorded at 5 second intervals between 5.08 and 5.42 min [N.B. Degassed water (30 mL) was added at a rate of 17.5 ml min^−1^ for all frames recorded between 5.08 and 6.50 min]. c) SAXS patterns recorded at 5 second intervals between 5.50 and 6.00 min, 30 second intervals between 6.00 and 9.50 min, and 1 min intervals between 9.50 and 20.50 min. d) SAXS patterns recorded at 1 min intervals between 21 and 50 min.

Selected SAXS patterns recorded during the TR‐SAXS experiment are shown in Figure 5 (see Supporting Information for further details, including data analysis protocols^[^ ^64^, ^65^ ^]^). The first frame was recorded after 1.75 min, which corresponds to the shortest possible delay owing to experimental constraints, i.e., the time taken for the reaction mixture to travel from the reaction vessel to the capillary flow cell (see purple pattern in Figure 5a). Rudimentary analysis of the low *q* region of this initial SAXS pattern using the well‐known Guinier equation [*I*(*q*) ∼ exp(‐*q* ^2^ *R*_go_^2^/3),^[^ ^66^ ^]^ where *q* is the scattering vector length and *R*_go_ is the radius of gyration of the scattering object] indicates a mean diameter (2*R*_go_) of approximately 223 nm. Bearing in mind the SAXS pattern and TEM image shown in Figure 4c,d, respectively, the original PCL_24_‐TTC droplets become appreciably smaller within 1.75 min. In principle, this size reduction could be the result of the formation of PCL_24_‐PDMAC_x_ diblock copolymer chains. However, the instantaneous DMAC conversion after 1.75 min is estimated to be only 1%. Alternatively, the difference in mean droplet diameter indicated by SAXS when comparing Figure 4c with the 1.75 min frame shown in Figure 5a could simply be the result of a difference in the shear history for these two samples.

**Figure 6:**
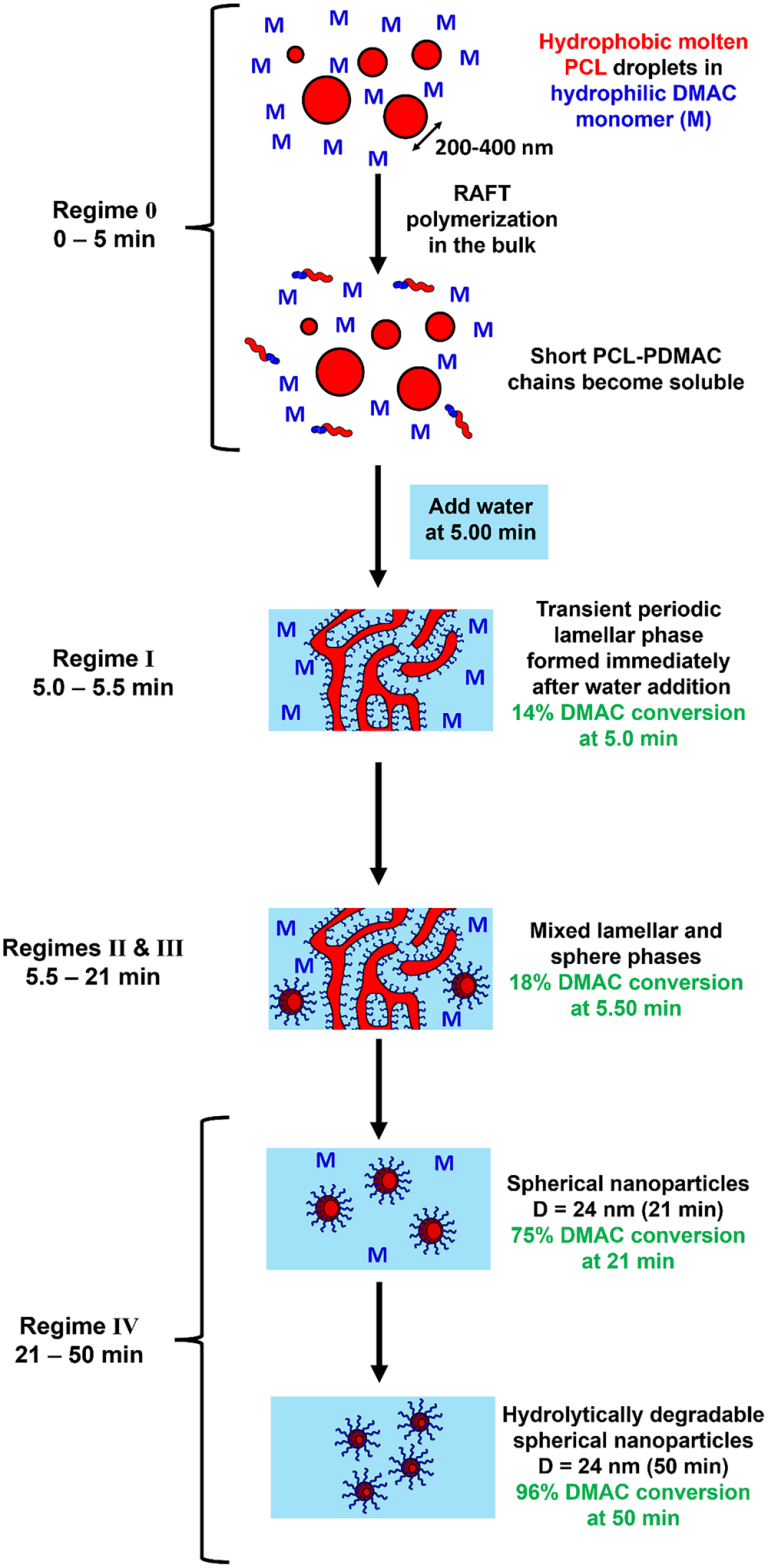
Schematic representation of the evolution of structure during *reverse sequence* aqueous PISA when targeting PCL_24_‐PDMAC_100_ nanoparticles. The initial reaction mixture is an optically transparent emulsion comprising molten PCL_24_‐TTC droplets dispersed in DMAC monomer (regime 0). Then RAFT polymerization of DMAC in the bulk at 80 °C begins to generate relatively short diblock copolymer chains. On addition of water to the reaction mixture after 5 min (14% DMAC conversion), the amphiphilic PCL_24_‐PDMAC_14_ chains undergo immediate self‐assembly to form a transient lamellar phase (regime I), which quickly evolves to form nascent spherical nanoparticles (regimes II to IV). As the DMAC polymerization proceeds within regime IV, such nanoparticles maintain their overall size while reducing their aggregation number to produce the final sterically stabilized spherical diblock copolymer nanoparticles (96% DMAC conversion).

As the DMAC polymerization progresses, an increase in X‐ray scattering intensity is observed at high *q* prior to the addition of water after 5.0 min (see Figure 5a). This is attributed to the growth of a relatively short PDMAC chain from one end of each PCL_24_ precursor, which leads to gradual dissolution of the resulting amphiphilic diblock copolymer chains within the continuous phase. After 5.0 min, water (30 mL) was added to the reaction mixture at a rate of 17.5 mL min^−1^. Thus, the overall time scale required for this water addition step is approximately 1.71 min. However, to simplify analysis of the resulting SAXS patterns, this water addition step is assumed to be effectively instantaneous. Although miscible with DMAC monomer, water is a non‐solvent for the hydrophobic PCL block, so its addition should lead to immediate self‐assembly. Indeed, time‐resolved SAXS patterns recorded shortly after water addition (5.08 min) confirm the appearance of a pronounced structure peak at *q* ∼ 0.025 Å, which indicates microphase separation (see Figure 5b). This feature is consistent with the formation of a periodic lamellar structure formed by the relatively asymmetric amphiphilic PCL_24_‐PDMAC_14_ chains (see inset cartoon in Figure 5b). This lamellar phase^[^ ^67^ ^]^—which indicates (either a planar bilayer or multilamellar vesicles—then rapidly evolves to produce nascent spherical nanoparticles, as indicated by the approximate zero gradient observed at q ∼ 0.01 Å^−1^ (see Figures 6 and 5c).^[^ ^66^ ^]^ Furthermore, the initial pronounced upturn in scattering intensity at low *q* disappears soon after water addition. This suggests the rapid transformation of the initial PCL_24_ emulsion droplets into a transient lamella phase, quickly followed by the formation of spherical nanoparticles (Figure 6; see regimes 0 to IV).

For the sake of simplicity, appropriate scattering models^[^ ^68^, ^69^, ^70^, ^71^, ^72^ ^]^ were only applied to those patterns recorded after water addition (see Equations S1–S9).

In principle, the evolution from the transient lamellar phase to the nascent spherical nanoparticles might be expected to involve both jellyfish and worms as transient intermediate structures.^[^ ^45^, ^67^, ^73^ ^]^ However, accounting for such structures would require a highly complex scattering model with many parameters, which would be unsuitable for reliable data fitting. Instead, the overall X‐ray scattering intensity was expressed as a sum of intensities originating from just lamellae and spheres; see Equation S1. To aid the data analysis, the *R*_g_ of the nanoparticles was calculated from the instantaneous PDMAC DP indicated by the DMAC conversion obtained from an ^1^H NMR kinetics study conducted using precisely the same reaction conditions as those employed for the TR‐SAXS experiment (see Figure 2). For the final frame (96% DMAC conversion), the mean contour length of the PDMAC_96_ coronal chains is estimated to be 24.5 nm (assuming that the length of two C–C bonds in an all‐*trans* configuration is 0.255 nm, hence 96 × 0.255 nm = 24.5 nm), while the corresponding Kuhn length is estimated to be around 1.53 nm (based on literature data for PMMA^[^ ^74^ ^]^). This suggests that the unperturbed radius of gyration (*R*_g_) for PDMAC_96_ is (24.5 nm × 1.53 nm/6)^0.5^ or approximately 2.5 nm. In addition, the volume fraction of water within the hydrophobic PCL_24_ cores, *x*_sol_, was calculated for the final frame recorded for the PCL_24_‐PDMAC_96_ nanoparticles after 50 min (see Figure S9 and the SAXS model described in the Supporting Information). An *x*_sol_ value of 0.12 was obtained, and this parameter was assumed to be constant regardless of the DMAC conversion. Accordingly, it was used to fit the scattering patterns recorded for the evolving spherical nanoparticles within regime IV.

Additional constraints were also derived by fitting this final SAXS pattern (see Figure S9). At this point, TEM analysis confirmed that only spherical nanoparticles were present (see Figure 4a). Fitting this final frame using a well‐known scattering model for spherical micelles^[^ ^75^ ^]^ indicated that the width of the micelle corona profile (see parameter *s* in Equation S5) should be close to three *R*_g_ for the PDMAC coronal block. According to the literature on spherical micelles, if the corona contribution dominates the scattering signal (see the SAXS model description in the Supporting Information), then *s* is typically 2.0 to 2.5‐times the *R*_g_ of the coronal chains.^[^ ^71^ ^]^ However, *s* may increase to more than three times *R*_g_ at elevated temperature,^[^ ^69^ ^]^ which is consistent with the SAXS data obtained for the final PCL_24_‐PDMAC_96_ nanoparticles at 80 °C. Given the relatively poor signal‐to‐noise ratio observed at high *q*, *s* was fixed at 3*R*_g_ instead of being used as a fitting parameter (see Equation S5). In addition, fitting the final frame recorded for the PCL_24_–PDMAC_96_ nanoparticles using the spherical micelle model indicates that the micelle coronal layer shape parameter, *a*, approaches zero, which suggests that the corresponding scattering length density profile is consistent with an extended brush conformation (see Figure S9). This is physically realistic for small nanoparticles comprising relatively long coronal blocks.^[^ ^76^ ^]^

**Figure 7:**
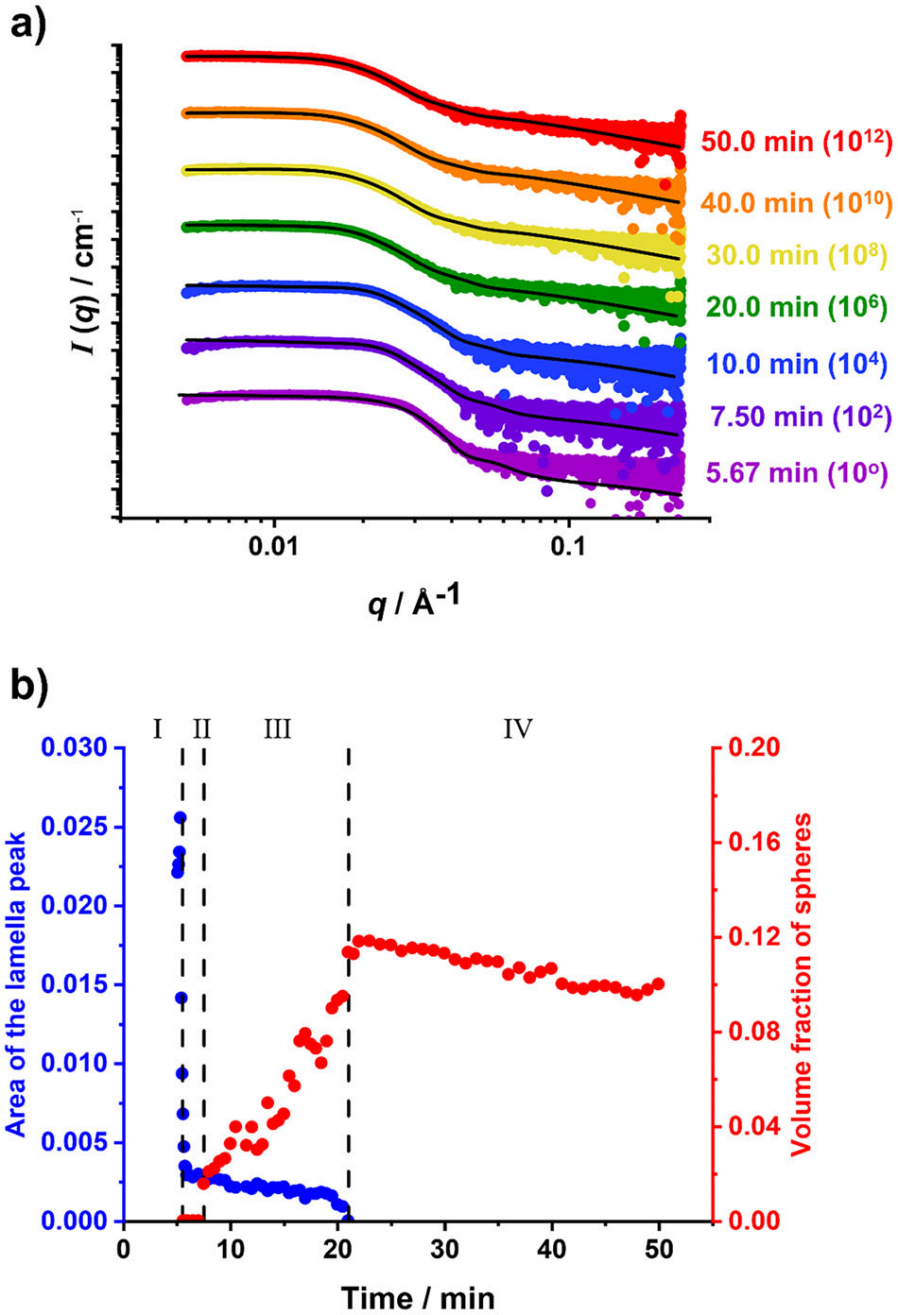
a) Arbitrarily offset SAXS patterns recorded during the synthesis of PCL_24_‐PDMAC_96_ spherical nanoparticles at 5.67 min (mauve frame), 7.50 min (purple frame), 10.0 min (blue frame), 20.0 min (green frame), 30.0 min (yellow frame), 40.0 min (orange frame), and 50.0 min (red frame). Solid black lines indicate the corresponding data fits. b) Plots of the volume fraction of spheres (red data) and area of the lamella peak (area of the Gaussian peak fitted to the lamellar diffraction peak, Equation S2; blue data) as a function of time calculated using a two‐population scattering model (see Equation S1).

Throughout the course of the DMAC polymerization, we make four assumptions regarding the relative volume fractions of the lamellae and sphere populations to analyze the scattering patterns (see Figure 7a). These assumptions correspond to regimes I to IV; see Figure 7b. In regime I (5.08 to 5.42 min), each SAXS pattern can be fitted assuming that solely lamellae are present, i.e., without requiring any scattering contribution from spherical nanoparticles.

Regime II (5.50 to 7.0 min) contains patterns that can be fitted by combining a lamellar structure factor with a spherical form factor. Inclusion of a spherical structure factor of increasing intensity (with a concomitant reduction in the lamellar scattering contribution) is required to fit the more complex scattering patterns recorded within regime III (7.50 to 20.5 min). Only spheres and lamellae are considered in regimes II and III, with alternative morphologies such as worms or jellyfish being deliberately excluded from the scattering model to avoid complexity. However, this simplified approach is likely to make data analysis less reliable.

In contrast, only a spherical nanoparticle population is required to produce satisfactory fits to the scattering patterns recorded within regime IV (21.0 to 50.0 min). Hence, this latter regime is considered the most reliable. Nevertheless, satisfactory data fits could be obtained for all scattering patterns recorded after water addition by making reasonable assumptions for each regime (see Figure 7a).

The pure lamellar phase (see Figure 5b) can be fitted using a Gaussian function (Equation S2) to account for the effective volume fraction of the lamellae. This structure peak remains prominent throughout regime I. In contrast, an additional population of spheres was required to achieve satisfactory data fits for patterns recorded between 5.50 and 7.0 min (regime II), as the lamellae structure peak becomes less prominent. However, no spherical structure factor was required because relatively few spheres are present at this time point. The volume fraction of spherical nanoparticles increases throughout regime III, so an additional structure factor for interacting spherical micelles is required to produce satisfactory data fits to the corresponding SAXS patterns. As more parameters are introduced into the scattering model, further constraints are desirable. For a moderately dilute dispersion of spherical nanoparticles, Pedersen and Gerstenberg reported that the shortest distance between neighboring cores with respective core radii, 2ΔR, is comparable to the width of their corona profiles, 2s.^[^ ^69^ ^]^ A similar conclusion can be drawn for the present study by fitting the SAXS pattern recorded for the final PCL_24_‐PDMAC_96_ spherical nanoparticles at the end of the reaction (see Figure S9). However, the high *q* regime for SAXS patterns recorded at intermediate DMAC conversion is relatively noisy (see Figure 5c). Hence the corresponding data were fitted using the scattering model (Equation S1) by imposing an additional constraint: Δ*R* = s. This condition corresponds to the onset of repulsive interactions between neighboring nanoparticles (see Figure S10).

This approach produced satisfactory fits for SAXS patterns recorded from 7.50 to 20.5 min; see Figure 7a. Finally, satisfactory data fits to all SAXS patterns recorded between 21.0 and 50.0 min could be obtained by assuming a single population of spherical nanoparticles; see Figure 7a.

**Figure 8:**
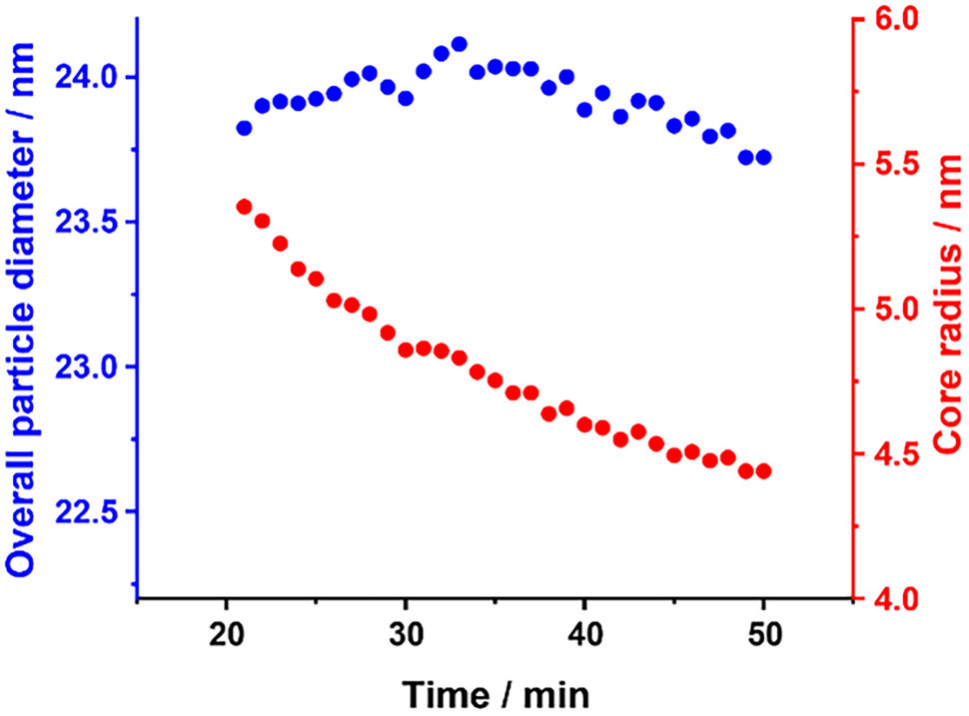
Evolution of the overall diameter (blue data) and mode core radius (red data) within regime IV during the synthesis of PCL_24_‐PDMAC_96_ spherical nanoparticles by *reverse sequence* aqueous PISA, as judged by TR‐SAXS studies.

Analysis of the scattering patterns recorded during regime IV required fewer assumptions and approximations than for regimes II and III. Thus the resulting data are considered more reliable (see Figure 8). Assuming that there is an equilibrium between the individual diblock copolymer chains and micelles/nanoparticles, the accepted theory^[^ ^77^ ^]^ for block copolymer self‐assembly predicts that increasing the steric stabilizer block DP for a given core‐forming block DP should result in a lower aggregation number and a concomitant reduction in size for the corresponding spherical micelles/nanoparticles. Moreover, this scaling law prediction is supported by recent SAXS data reported by Warren and coworkers.^[^ ^78^ ^]^ On the other hand, if there is little or no exchange of diblock copolymer chains between the nanoparticles on the time scale of the DMAC polymerization studied herein (i.e., if the system is non‐ergodic and the PCL cores are frozen),^[^ ^79^, ^80^ ^]^ then the nanoparticle core radius and aggregation number should remain constant while the overall nanoparticle diameter should *increase* as the PDMAC stabilizer chains grow longer.

Inspecting Figure 8, the overall nanoparticle diameter remains constant at approximately 24 nm from 21 to 40 min, with an apparent marginal reduction of ∼ 0.3 nm between 40 and 50 min. Moreover, SAXS analysis indicates a reduction in the nanoparticle core radius from 5.4 to 4.4 nm within regime IV, with a concomitant reduction in the aggregation number from 119 to 68 being observed over the same time scale (see Figure S11). Thus, SAXS analysis suggests that there must be an equilibrium between the spherical nanoparticles and the individual diblock copolymer chains during the DMAC polymerization, which enables the nanoparticle cores to adjust their size to accommodate the growing coronal chains. This is consistent with partial plasticization of the core‐forming PCL_24_ chains (*x*_sol_ = 0.23) indicated by SAXS. Moreover, it seems likely that the reduction in the spherical micelle volume fraction observed in Figure 7b is the result of a gradual increase in the relative concentration of the molecularly dissolved copolymer chains driven by continuous growth of the hydrophilic PDMAC block.

Despite the experimental uncertainties, the scattering patterns recorded for regimes I, II, and III were fitted using the two‐population model comprising solely lamellae and spheres. When spherical nanoparticles first appear at 5.50 min, SAXS analysis indicates an overall nanoparticle diameter of 21 ± 5 nm, which increases to 24 ± 7 nm at the end of regime III (see Figure S11a). Moreover, a significant reduction in the mode nanoparticle core radius is observed from 7.4 nm (regime II) to 5.4 nm (regime III); see Figure S11b. This is accompanied by a corresponding reduction in the aggregation number from 319 to 119 (see Figure S11c).

### Combining Reverse Sequence PISA with CDSA to Produce PCL_24_‐PDMAC_74_ Rods at 15 °C

**Figure 9:**
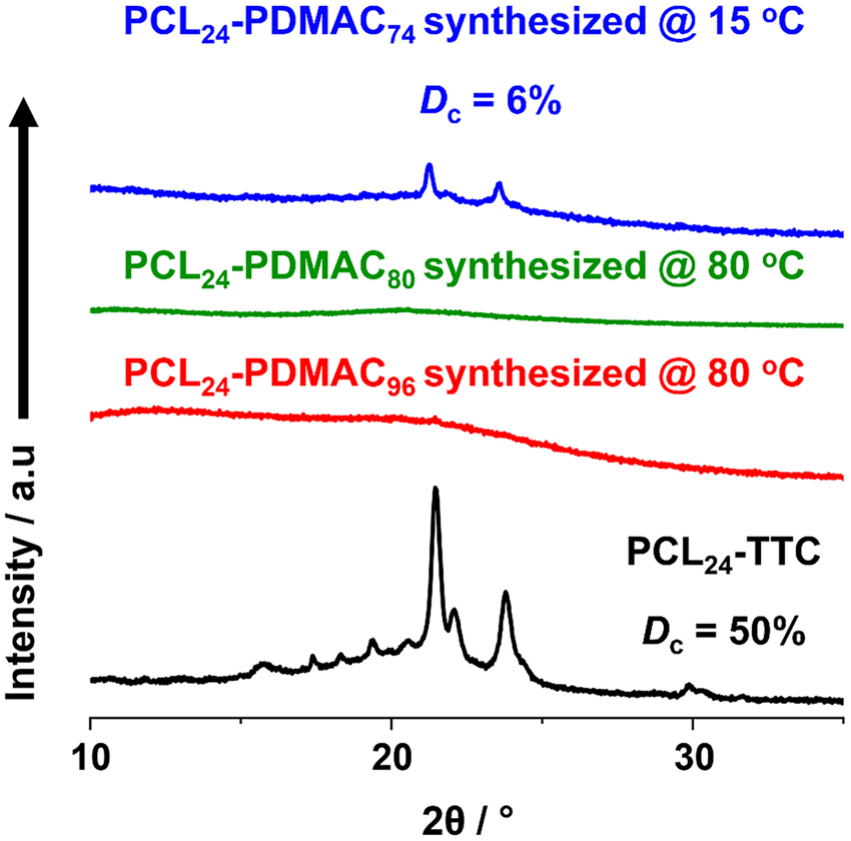
XRD patterns recorded for the PCL_24_‐TTC precursor (black curve), freeze‐dried PCL_24_‐PDMAC_96_ spherical nanoparticles prepared at 80 °C during the TR‐SAXS experiment (red curve), freeze‐dried PCL_24_‐PDMAC_80_ spherical nanoparticles prepared at 80 °C (green curve), and freeze‐dried PCL_24_‐PDMAC_74_ rod‐like nanoparticles prepared at 15 °C (blue curve). See main text for further details.

It is well known that semicrystalline PCL‐based diblock copolymers can undergo CDSA after aging at ambient temperature to form either rods and/or platelets, respectively.^[^ ^33^, ^34^, ^35^, ^36^, ^81^ ^]^ On the other hand, our *reverse sequence* aqueous PISA formulation produces solely spherical nanoparticles if the DMAC polymerization is performed at 80 °C when targeting PCL_24_‐PDMAC_100_. This is simply because this reaction temperature exceeds the *T*_m_ of 45 °C for the PCL block. Moreover, crystallization of the amorphous cores would not be expected to occur on cooling to 20 °C owing to the nanoscale confinement of the PCL chains. The critical nucleus size for homogeneous nucleation, l*, is given by l* = 4*σ*_e_.(*T* ^0^_m_ + 273)/(Δ*T*.Δ*H*_m_.*ρ*_c_),^[^ ^82^ ^]^ where the surface free energy for chain‐folding, *σ*_e_ is 106 mJ m^−2^, the equilibrium melting temperature, *T* ^0^_m_ is 75 °C, the latent heat of fusion at the equilibrium melting temperature, Δ*H*_m_ is 148 J g^−1^, Δ*T* = *T* ^0^_m_ – *T*_c_ is the degree of undercooling, *T*_c_ is the crystallization temperature, and the density of the PCL crystals, *ρ*_c_ is 1.187 g cm^−3^.^[^ ^83^ ^]^ If nucleation takes place at 20 °C, then Δ*T* = 55 °C. For such undercooling, the critical minimum size for homogeneous PCL crystalline nuclei is calculated to be around 15 nm, which is significantly larger than the mean core diameter of 8.8 nm observed for the final spherical nanoparticles (see Figure S9). Furthermore, this mean core diameter is also less than the period of the lamellar phase (9.7 nm) formed by the phase‐separated amorphous and crystalline domains of the semicrystalline PCL_24_ homopolymer in the solid state (see Figure S12). In summary, these observations explain why the nanoparticles formed at 80 °C retain their amorphous PCL cores after cooling to 20 °C. Accordingly, the PCL_24_‐TTC precursor and the freeze‐dried PCL_24_‐PDMAC_96_ nanoparticles produced during the TR‐SAXS experiment were subjected to X‐ray diffraction (XRD) analysis. The XRD plot (see black curve in Figure 9) obtained for the PCL_24_‐TTC homopolymer contains multiple Bragg peaks, and its degree of crystallinity, *D*_c_, is calculated to be approximately 50%. This is consistent with the corresponding SAXS pattern (Figure S12), which indicates that the semicrystalline PCL_24_‐TTC chains form a lamellar phase. On the other hand, the XRD plot recorded for the corresponding PCL_24_‐PDMAC_96_ spherical nanoparticles is featureless, with no evidence for any Bragg peaks (see red curve in Figure 9). Clearly, CDSA does not occur during their synthesis at 80 °C.

**Figure 10:**
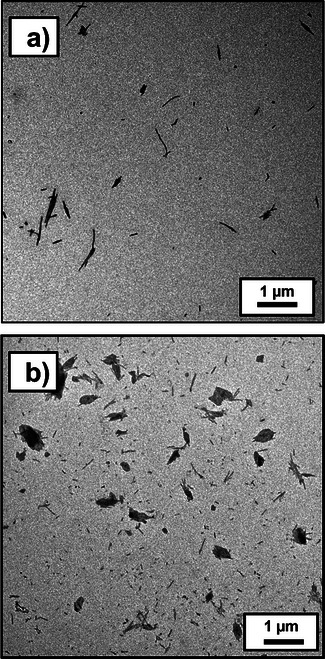
a) Representative TEM images recorded for 0.1% w/w aqueous dispersion of (a) rod‐like PCL_24_‐PDMAC_74_ nanoparticles (*D*_c_ = 6%) at 15 °C (see entry 2 in Table S1) and (b) partially aggregated rod‐like PCL_24_‐PDMAC_55_ nanoparticles synthesized at 15 °C (see entry 1 in Table S1).

To combine this *reverse sequence* aqueous PISA formulation with CDSA, the DMAC polymerization must be performed below the *T*_m_ of 45 °C for the PCL block. Accordingly, the UV‐initiated polymerization of DMAC (*λ* = 365 nm) was conducted at 15 °C using the same PCL_24_ precursor as that employed for the TR‐SAXS experiment (see Figure 1b), with AIBN acting as a photoinitiator.^[^ ^84^, ^85^ ^]^ Preliminary experiments were performed in the absence of any AIBN initiator. However, such photoiniferter polymerizations^[^ ^86^ ^]^ were relatively slow and proved to be unsuccessful: water addition was not possible within a reasonable time frame (<4 h) without causing macroscopic precipitation. In view of this problem, AIBN was added to produce a faster polymerization. The rate of polymerization was still significantly slower than the equivalent thermally initiated polymerization conducted at 80 °C. Nevertheless, water was added after 3.5 h (intermediate DMAC conversion = 26%; PDMAC DP ∼ 21) to target PCL_24_‐PDMAC_80_ nanoparticles at 30% w/w solids. Gratifyingly, ^1^H NMR spectroscopy studies indicated that a final DMAC conversion of 93% was achieved after 24 h of continuous UV irradiation (see Figure S13). GPC analysis indicated the formation of well‐defined, low‐dispersity diblock copolymer chains (see Figure S14). Importantly, XRD studies confirmed that the PCL cores of these nanoparticles were indeed semicrystalline (*D*_c_ = 6%), unlike when the same DMAC polymerization was performed at 80 °C (compare blue and green curves in Figure 9). Initially, a DP of 100 was targeted for the AIBN‐initiated polymerization of DMAC using the PCL_24_‐TTC precursor via UV irradiation at 15 °C. However, the XRD pattern recorded for the resulting PCL_24_‐PDMAC_93_ diblock copolymer indicated only 3% crystallinity (see Figure S15). Moreover, only ill‐defined aggregates were produced rather than well‐defined rods (see Figure S16). Reducing the target PDMAC DP to 80 increased the mean degree of crystallinity to 6% (see Figure S15). The corresponding TEM images suggest that targeting a shorter PDMAC DP affords more well‐defined rod‐like nanoparticles (see Figure 10a). When the target PDMAC DP was reduced to 60, the mean degree of crystallinity was 11%, but the rod‐like nanoparticles were obtained in the form of aggregates or “rafts” (see Figures 10b and S17).

Unfortunately, ultrasonic treatment of such aggregates for 3 h did not improve their degree of dispersion. This is quite surprising, as this protocol has been reported to break up relatively large rods formed by CDSA into smaller seeds.^[^ ^27^, ^35^ ^]^

When targeting a PDMAC DP of 80, a final DMAC conversion of 93% was achieved after continuous UV irradiation at 15 °C for 24 h. The polymerization temperature was increased to 30 °C to target a higher conversion. Indeed, more than 99% DMAC conversion was achieved within 24 h under such conditions. However, XRD studies indicated zero crystallinity for the final diblock copolymer nanoparticles (see Figure S18). Moreover, only ill‐defined aggregates could be observed by TEM (see Figure S19a), which are similar to those observed when targeting a PDMAC DP of 100 at 15 °C (see Figure S16). In a second experiment, the synthesis of PCL_24_‐PDMAC_x_ (*x* = 80) nanoparticles was attempted at 10 °C. In this case, XRD indicated a *D*_c_ of 8% (see Figure S18), but chain extension was relatively inefficient, and the diblock copolymer chains exhibited a relatively broad molecular weight distribution (see Figure S20). TEM analysis indicated a mixture of rod‐like nanoparticles and platelets (see Figure S19b). It is well known that adding PCL homopolymer while conducting traditional CDSA processing of PCL‐PDMAC diblock copolymers in dilute solution can produce platelets.^[^ ^33^ ^]^ Thus the residual PCL_24_ precursor clearly visible in the GPC curve recorded for the PCL_24_‐PDMAC_80_ diblock copolymer most likely accounts for the platelet population observed by TEM.

**Figure 11:**
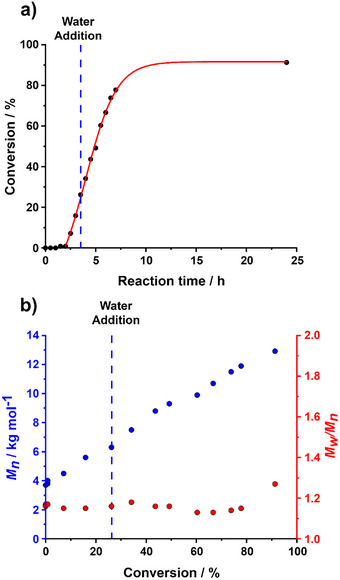
Kinetics of polymerization observed when preparing PCL_24_‐PDMAC_80_ nanoparticles via *reverse sequence* PISA combined with CDSA, in which the DMAC polymerization is initiated in the bulk at 15 °C under continuous UV irradiation (*λ* = 365 nm) followed by dilution of the reaction mixture with degassed deionized water after 3.5 h (DMAC conversion = 26%) to target 30% w/w solids. Conditions: [PCL_24_‐TTC]/[AIBN] molar ratio = 1.0. a) Conversion versus time curve (black data) obtained by ^1^H NMR spectroscopy studies of the periodically sampled reaction mixture and b) the corresponding evolution of *M*_n_ (blue data) and *M*_w_/*M*_n_ (red data) with conversion as determined by DMF GPC analysis (expressed relative to a series of near‐monodisperse PMMA calibration standards).

In summary, optimal conditions for the synthesis of PCL_24_‐PDMAC_x_ rod‐like nanoparticles at 30% w/w solids require a PDMAC DP of 80 to be targeted at 15 °C. Accordingly, a kinetic study was conducted for this formulation with periodic sampling of the reaction mixture during continuous UV irradiation. A final DMAC conversion of 93% was again achieved within 24 h (see Figure 11a), and the growing diblock copolymer chains exhibited relatively low dispersities throughout the polymerization (see Figure 11b). Although beyond the scope of the current study, it seems likely that the UV irradiation efficiency of our homemade UV cell could be significantly improved. In principle, this should enable faster DMAC polymerizations (and higher final DMAC conversions) to be achieved. One reviewer of this manuscript asked whether such rod‐like nanoparticles might have any specific applications. In addition to their potential use as Pickering emulsifiers, one possible application could be a hydrolytically degradable thickener for aqueous formulations. However, this would most likely require the synthesis of significantly more anisotropic rods than those reported herein.^[^ ^87^ ^]^

## Conclusions

The *reverse sequence* PISA synthesis of PCL_24_‐PDMAC_x_ nanoparticles in aqueous media has been revisited. A time‐resolved SAXS experiment has been performed when conducting this synthesis at 80 °C while targeting *x* = 100. Under these conditions, the final nanoparticles obtained at 96% DMAC conversion possess a spherical morphology, a nanoparticle core radius of 4.4 nm, and a corresponding aggregation number of 68. SAXS analysis reveals that the PCL_24_ precursor is actually present as molten droplets within the DMAC monomer, rather than being truly molecularly dissolved as originally reported.^[^ ^41^ ^]^ This misunderstanding arose because of the very small difference in refractive index between the PCL and the DMAC monomer at 80 °C, which fortuitously leads to a highly transparent emulsion. The addition of water at an intermediate DMAC conversion of 14% initially produces a transient lamellar phase, which rapidly evolves to form nascent spherical nanoparticles (up to a final DMAC conversion of 96%). Interestingly, there is a subtle concomitant *reduction* in both the PCL core diameter and the aggregation number as the PDMAC steric stabilizer chains grow longer, which is consistent with the known scaling laws for block copolymer self‐assembly in solution.^[^ ^77^, ^78^ ^]^ This observation implies that there must be rapid exchange between the nanoparticles and the individual diblock copolymer chains during the DMAC polymerization. On cooling to 20 °C, the nanoparticles retain their spherical morphology, and the PCL cores remain amorphous. This is simply because confinement prevents PCL crystallization: the nanoparticle core diameter of approximately 8.8 nm is less than the critical minimum size of approximately 15 nm required for the formation of stable crystalline PCL nuclei during homogeneous nucleation. In contrast, if UV irradiation is used to conduct the DMAC polymerization at 15 °C (i.e., well below the *T*_m_ of 45 °C for the PCL chains), this alternative *reverse sequence* aqueous PISA formulation produces rod‐like nanoparticles when targeting PCL_24_‐PDMAC_80_. This is because the hydrophobic PCL chains form semicrystalline cores under such conditions, so nanoparticle formation is governed by CDSA. This is confirmed by XRD analysis, which indicates a mean degree of crystallinity of 6% for the dried rods (versus 50% for the PCL_24_‐TTC precursor). This study provides important new physical insights regarding the complex mechanism of nanoparticle formation during *reverse sequence* aqueous PISA while further exemplifying the versatility of such formulations for the preparation of concentrated aqueous dispersions of either spherical or rod‐like hydrolytically degradable nanoparticles.

## Conflict of Interests

The authors declare no conflict of interest.

## Supporting information



Supporting information

## Data Availability

The data that support the findings of this study are available in the Supporting Information of this article.
