# Supplementary material for: Time‐Resolved SAXS Studies During the Synthesis of Hydrolytically Degradable Poly(ε‐caprolactone)‐Poly(N,N′‐dimethylacrylamide) Diblock Copolymer Nanoparticles in Aqueous Media
Source: Angew Chem Int Ed Engl. 2025 Nov 17;65(2):e24000. doi: 10.1002/anie.202524000 (PMC12790348; doi:10.1002/anie.202524000)
Supplement: Supplementary file 1 — Supporting information [file ANIE-65-e24000-s001.docx]

Supporting Information for:

***Time-resolved SAXS studies during the synthesis of hydrolytically degradable poly(ε-caprolactone)-poly(N,N’-dimethylacrylamide) diblock copolymer nanoparticles in aqueous media***

Matthew A. H. Farmer^a^, Oleksandr O. Mykhaylyk,^a^ Olga Shebanova,^b^ Osama M. Musa^c^ and Steven P. Armes^a^

*a. Dainton Building, School of Mathematics and Physical Sciences, University of Sheffield, Brook Hill, Sheffield, South Yorkshire, S3 7HF, UK.*

*b. Diamond Light Source Ltd, Diamond House, Harwell Science and Innovation Campus, Didcot, Oxfordshire OX11 0DE, UK.*

*c. Ashland Specialty Ingredients, 1005 US 202/206, Bridgewater, New Jersey, 08807, USA.*

Contents

[**Experimental** S2](#_Toc191904981)

[Materials S2](#_Toc191904982)

[Characterization Techniques S2](#_Toc191904983)

[Synthetic Protocols S7](#_Toc191904984)

[**Additional Characterization Data** S9](#_Toc191904985)

[**References** S19](#_Toc191904986)

#

# **Experimental**

##

## **Materials**

All reagents were used as received, unless stated otherwise. 4,4′-Azobis(4-cyanopentanoic acid) (ACVA; 98%), dicyclohexylcarbodiimide (DCC; 99%), 2,2′-azobis(2-methylpropionitrile) (AIBN; 98%), *N*,*N’*-dimethylacrylamide (DMAC; 99%), triazabicyclodecene (TBD), anhydrous magnesium sulfate, lithium bromide, calcium hydride, ε-caprolactone and benzyl alcohol were purchased from Sigma-Aldrich (Dorset, UK). The latter two reagents were dried over calcium hydride and distilled before use. AIBN was recrystallized in methanol prior to use. 4-(dimethylamino)pyridine (DMAP) was purchased from Alfa Aesar (Heysham, UK). Benzoic acid was purchased from Fluorochem Limited (Hadfield, UK). *N,N’*-Dimethylformamide (DMF) was purchased from VWR (Leicestershire, UK). Deuterated dichloromethane (99.8%) was purchased from Goss Scientific Instruments Ltd. (Cheshire, UK). Anhydrous dichloromethane and toluene were obtained from an in-house Grubbs purification solvent system. 4-Cyano-4-(ethylsulfanylthiocarbonyl)sulfanylpentanoic acid (CEPA) and the corresponding poly(ε-caprolactone)-TTC precursor were prepared using a literature protocol.^1,2^ Deionized water was dispensed from an Elgastat Option 3A water purification system with a resistivity of 15 MΩ cm.

##

## **Characterization Techniques**

**^1^H Nuclear Magnetic Resonance Spectroscopy.**  Spectra were recorded using a 400 MHz Bruker Avance-400 spectrometer operating at 298 K with 16 scans being averaged per spectrum. Samples were dissolved in CD_2_Cl_2_ and aqueous copolymer dispersions were dried with anhydrous magnesium sulfate before passing through a 0.20 µm filter. DMAC conversions were calculated by comparing the integrated vinyl proton signals at 6.63, 6.23 and 5.66 ppm with the 4.1 ppm signal assigned to the COO-CH_2_ ester protons on the PCL block.

**Gel Permeation Chromatography.** An Agilent 1260 Infinity GPC system equipped with a differential refractive index detector and a UV detector set at 305 nm was used to determine the number-average molecular weight (*M*_n_), weight-average molecular weight (*M*_w_) and dispersity (*M*_w_/*M*_n_) for each (co)polymer. Two Agilent PL-gel 5 μm Mixed-C columns and a guard column were connected in series to this GPC instrument. HPLC-grade DMF containing 10 mM LiBr was used as the eluent. GPC analysis was performed at 60 °C using a constant flow rate of 1.0 mL min^−1^. A series of twelve near-monodisperse poly(methyl methacrylate) calibration standards with *M_p_* values (ranging from 800 g mol^-1^ to 2 200 000 g mol^-1^) was used to calculate molecular weights and dispersities. All (co)polymer samples were diluted to 1.0% w/w using DMF and chromatograms were analyzed using Agilent GPC/SEC software.

**Dynamic Light Scattering.** Experiments were conducted at 20 °C using a Malvern Instruments Zetasizer Nano ZS instrument equipped with a 4 mW He−Ne laser (λ = 633 nm). Scattered light was detected at 173 ° with an avalanche photodiode detector. Aqueous block copolymer dispersions were diluted to 1.0% w/w with deionized water prior to analysis. Five minutes was allowed for thermal equilibration at the beginning of each measurement. The mean z-average particle diameter (*D_z_*) and polydispersity index (PDI) were averaged over three consecutive runs consisting of ten measurements each.

**Transmission Electron Microscopy (TEM).** Copper grids (Agar Scientific, UK) were coated in-house with a thin film of amorphous carbon and then treated with a plasma glow discharge for 30 seconds to generate a hydrophilic surface. An 8 μL droplet of freshly diluted 0.1% w/w aqueous copolymer dispersion was placed on a hydrophilic grid for 1 min, then blotted to remove excess sample. Each grid was negatively stained for a further 30 seconds using an 8 μL droplet of 0.75% w/v aqueous uranyl formate solution, which was then carefully blotted to remove excess stain. Each grid was dried with the aid of a vacuum hose. Imaging was performed using a FEI Tecnai Spirit 2 microscope equipped with an Orius SC1000B camera operating at 80 kV. The same protocol was employed when imaging the initial PCL_24_-TTC droplets: DMAC monomer was used to dilute the PCL_24_-TTC emulsion to 0.1% w/w, prior to heating up to 80 °C to prepare the TEM grid.

**Small-Angle X-ray Scattering Studies (SAXS).** SAXS experiments were conducted on a 25% w/w emulsion of PCL_24_–TTC in DMAC monomer at the ESRF (station ID02, Grenoble, France)^3^ using monochromatic X-ray radiation [*λ* = 1.03 Å; *q* range = 0.0008 to 0.08 Å^–1^, where *q* is the length of the scattering vector and *θ* is one-half of the scattering angle, such that *q* = (4π/*λ*).sin*θ*] and a Eiger2 4M hybrid pixel two-dimensional (2D) detector (Dectris, Switzerland). A flow-through glass capillary of 1.72 mm diameter was used as a sample holder and heated to 80 ^o^C. The emulsion was allowed to equilibrate for 2 min in this sample holder prior to recording ten SAXS patterns, which were then averaged prior to analysis. The SAXS pattern for the solid PCL_24_-TTC precursor was recorded using a Xeuss 2.0 laboratory beamline (Xenocs, Grenoble, France) equipped with a Pilatus 1M detector (Dectris, Baden, Switzerland) and a Xenocs Genix3D X-ray source (CuKα radiation, λ = 1.54 Å; *q* range = 0.008 to 0.6 Å^–1^). Scattering data were reduced using XSACT software package supplied with the instrument.

A time-resolved SAXS study was performed at Diamond Light Source (station I-22, Didcot, UK)^6^ using monochromatic X-ray radiation [λ = 1.24 Å; *q* range = 0.002 to 0.2 Å^–1^] and a 2D Pilatus 2M hybrid pixel detector (Dectris, Switzerland). In this case, a 4.5 % w/w dispersion of PCL_24_-PDMAC_100_ nanoparticles was targeted. A PCL_24_-TTC precursor (0.84 g, 0.272 mmol), DMAC monomer (2.70 g; 27.2 mmol; target DP = 100) and ACVA initiator (15.2 mg; 0.054 mmol) were added to a 28 mL glass vial, which was sealed and purged with nitrogen gas for 30 min. With the outlet needle and nitrogen gas inlet needle still inserted, a third needle connected to Teflon tubing (15 cm length, 0.50 mm diameter) was inserted into the reaction flask (**Figure 3**). The other end of the tubing was connected to a 1.79 mm diameter flow-through capillary cell placed in line with the synchrotron X-ray source. This cell was connected to a motorized Harvard Apparatus PHD ULTRA™ Satellite Syringe Pump by Teflon tubing with the pump controller being placed outside the experimental hutch. A fourth needle was inserted into the vial and connected to a syringe of degassed water that had been heated to 80^o^C in an identical remote controlled syringe pump (**Figure 3**). The reaction vessel was then immersed in a preheated oil bath set at 80 °C and the reaction mixture was magnetically stirred. The syringe pump was set to a withdrawal rate of 0.50 mL min^–1^ for the first 5 min. Sampling commenced at a reaction time of 0.75 min. After 5 min, the withdrawal rate was increased to 2.0 mL min^-1^ and simultaneously water (30 mL) was gradually added to the reaction vessel at a rate of 17.5 mL min^-1^. After 7 min, the withdrawal rate was reduced to 1.00 mL min^-1^. The withdrawal rate was further reduced to 0.50 ml min^-1^ after 20 min and to 0.25 mL min^-1^after 30 min until the end of the reaction. The dead time for this experimental set-up is approximately 100 s, which corresponds to the time taken for the pumped reaction mixture to first reach the flow-through cell (see **Figure 3**). Scattering data were reduced using DAWN software with standard routines provided by the beamline.^4^ All the reduced scattering data were further analyzed using Irena SAS macros for Igor Pro.^5^

**SAXS Models**. The X-ray scattering intensity for the model used for in situ SAXS analysis is defined by the sum of the scattering from the lamellae and the spherical micelles (or nanoparticles):

| $I\left(q\right)=I_{\text{sm}}\left(q\right)+I_{\text{lam}}\left(q\right)$ | (S1) |
| --- | --- |
|  |  |

*I*_lam_(*q*) in **Equation S1** is represented by a Gaussian peak function:

| $I_{\text{lam}}\left(q\right)=I_{\max}\exp\left[-\frac{1}{2}\left(\frac{q-q_{\text{lam}}}{\sigma_{\text{lam}}}\right)^{2}\right]$, | (S2) |
| --- | --- |

where *q*_lam_ is the peak maximum position, *σ*_lam_ is the full-width at half maximum of the peak and *I*_max_ is the maximum intensity at the peak’s centre.

*I*_sm_(*q*) in **Equation S1** is represented by:

| , | (S3) |
| --- | --- |

assuming that the spherical micelles interact only with each other, and that any spherical micelle of a given size is always surrounded by micelles of the same size. This enables a local monodisperse approximation to be used^7^ to simplify calculations of the scattering intensity originating from spherical micelles. In **Equation S3**,  denotes the volume fraction of spherical micelles. Assuming that there is no penetration of the coronal blocks (PDMAC) within the micelle cores (PCL), the spherical micelle form factor in **Equation S3** can be expressed as:^8^

| $\begin{matrix} F_{\text{sm}}\left(q,r_{\text{mc}}\right)=n_{\text{agg}}^{2}\beta_{\text{mc}}^{2}A_{\text{mc}}^{2}\left(q,r_{\text{mc}}\right)+n_{\text{agg}}\left(n_{\text{agg}}-1\right)\beta_{\text{c}}^{2}A_{\text{c}}^{2}\left(q\right) \\ +n_{\text{agg}}\beta_{\text{c}}^{2}F_{\text{c}}\left(q,R_{\text{g}}\right)+2n_{\text{agg}}^{2}\beta_{\text{mc}}\beta_{\text{c}}A_{\text{mc}}\left(q,r_{\text{mc}}\right)A_{\text{c}}\left(q\right) \end{matrix}$, | (S4) |
| --- | --- |

where *r*_mc_ is the spherical micelle core radius and *R*_g_ is the radius of gyration of the PDMAC corona block. The X-ray scattering length contrasts for the PCL core block and the PDMAC corona block are $\beta_{\text{mc}}=V_{\text{mc}}\left(\xi_{\text{mc}}-\xi_{\text{sol}}\right)$and $\beta_{\text{c}}=V_{\text{c}}\left(\xi_{\text{c}}-\xi_{\text{sol}}\right)$, respectively. *ξ*_mc_, *ξ*_c_, and *ξ*_sol_ are X-ray scattering length densities for the core block (*ξ*_PCL_ = 10.05 × 10^10^ cm^-2^), corona block (*ξ*_PDMAC_ = 10.72 × 10^10^ cm^-2^) and the solvent (*ξ*_H2O_ = 9.42 × 10^10^ cm^-2^), respectively. *V*_mc_ and *V*_c_ are the volumes of the core block and the corona block, respectively. The block volumes can be calculated using the corresponding block molecular weight and mass density (*ρ*_PCL_ = 1.09 g cm^-3^ and *ρ*_PDMAC_ = 1.16 g cm^-3^), $V=\frac{M\cdot DP}{N_{\text{A}}\rho}$, where *M* is the molar mass of the repeat units within the block, *DP* is the mean degree of polymerization of the block, and *N*_A_ is Avogadro’s constant. The solid-state density of the semicrystalline PCL_24_-TTC homopolymer was determined by helium pycnometry, while the density of PDMAC homopolymer was taken from the literature.^9^ The mean aggregation number, *n*_agg_, in **Equation S4** is given by the expression $n_{\text{agg}}=\left(1-x_{\text{sol}}\right)\frac{4\pi r_{\text{mc}}^{3}}{3V_{\text{mc}}}$, where *x*_sol_ is the volume fraction of solvent (water) within the core.

The amplitude of the micelle core self-term in **Equation S4** is $A_{\text{mc}}\left(q,r_{\text{mc}}\right)=\Phi\left(qr_{\text{mc}}\right)\exp\left(-\frac{q^{2}\sigma_{\text{int}}^{2}}{2}\right)$, where $\Phi\left(qr_{\text{mc}}\right)=\frac{3\left[\sin\left(qr_{\text{mc}}\right)-qr_{\text{mc}}\cos\left(qr_{\text{mc}}\right)\right]}{\left(qr_{\text{mc}}\right)^{3}}$is the form factor amplitude of a sphere. The exponent term represents a sigmoidal interface between the blocks with a width *σ*_int_ and a decaying scattering length density at the core surface. During data fitting, this parameter was fixed at 2.5 Å, which corresponds to the length of two C-C bonds projected onto the polymer backbone. The self-correlation term for the corona block used in **Equation S4** is given by the Debye function: $F_{\text{c}}\left(q,R_{\text{g}}\right)=\frac{2\left[\exp\left(-q^{2}R_{\text{g}}^{2}\right)-1+q^{2}R_{\text{g}}^{2}\right]}{q^{4}R_{\text{g}}^{4}}$. For the final PCL_24_-PDMAC_96_ diblock copolymer composition, the PDMAC corona contribution to the scattering signal dominates the scattering arising from the PCL core [$(\beta_{\text{c}}/\beta_{\text{mc}})^{2}$≈ 6.74]. Thus, the amplitude of the corona chain form factor was obtained from a normalized Fourier transform of the radial density distribution function for the PDMAC corona chains:

| $A_{\text{c}}\left(q\right)=\frac{\int_{R_{\text{mc}}}^{R_{\text{mc}}+2s} \mu_{\text{c}}\left(r\right)\frac{\sin\left(qr\right)}{qr}r^{2}dr}{\int_{R_{\text{mc}}}^{R_{\text{mc}}+2s} \mu_{c}\left(r\right)r^{2}dr}\exp\left(-\frac{q^{2}\sigma_{\text{int}}^{2}}{2}\right)$, | (S5) |
| --- | --- |

where the radial profile, *μ*_c_(*r*), is expressed by a linear combination of two cubic b splines with two fitting parameters *s* and *a*, which correspond to the width of the micelle corona profile and the weight coefficient associated with the shape of the micelle corona blocks, respectively. *R*_mc_ is the mode value of the micelle core radius. Further information can be found elsewhere,^8^ as can the approximate integrated expression for **Equation S5**. The form factor for the average radial scattering length density distribution for spherical micelles indicated in **Equation S3** is given by:

| $F_{\text{sm}}^{\text{av}}\left(q,r_{\text{mc}}\right)=A_{\text{sm}}^{2}\left(q,r_{\text{mc}}\right)=n_{\text{agg}}^{2}\beta_{\text{mc}}^{2}A_{\text{mc}}^{2}\left(q,r_{\text{mc}}\right)+2n_{\text{agg}}^{2}\beta_{\text{mc}}\beta_{\text{c}}A_{\text{mc}}\left(q,r_{\text{mc}}\right)A_{\text{c}}\left(q\right)+n_{\text{agg}}^{2}\beta_{\text{c}}^{2}A_{\text{c}}^{2}\left(q\right)$. | (S6) |
| --- | --- |

The dispersity of the micelle core radius used in **Equation S3** is described by Schultz-Zimm distribution function commonly used for polymer particles:^10^

| $\Psi\left(r_{\text{mc}}\right)=\frac{\left(z/R_{\text{mc}}\right)^{z+1}r_{\text{mc}}^{z}}{\Gamma\left(z+1\right)}\exp\left[-zr_{\text{mc}}/R_{\text{mc}}\right]$, | (S7) |
| --- | --- |

which is normalized such that $\int_{0}^{\infty} \Psi\left(r_{\text{mc}}\right)dr_{\text{mc}}=1$. Here *z* is the distribution shape parameter, $\Gamma\left(z+1\right)$ is the gamma function. The standard deviation of *R*_mc_ is expressed as:

| $\sigma_{\text{R}_{\text{mc}}}=R_{\text{mc}}\frac{\left(1+z\right)^{1/2}}{z}$. | (S8) |
| --- | --- |

The structure factor term in **Equation S3** corresponding to inter-micelle interactions is described using the hard-sphere structure factor solved by the Percus-Yevick closure relation^11^ and is commonly used for the scattering analysis of spherical diblock copolymer micelles:^12^

| $S\left(q,r_{\text{mc}}\right)=S_{\text{PY}}\left(q,r_{\text{sf}},V_{\text{sf}}\right)$, | (S9) |
| --- | --- |

where *V*_sf_ is the effective volume fraction of the interacting micelles, the mean inter-micelle distance is defined as $2r_{\text{sf}}=2\left(r_{\text{mc}}+\Delta R\right)$, and $2\Delta R$ is the shortest distance between interfaces of the neighbouring micelle cores with respective core radius $r_{\text{mc}}$.

**X-Ray Diffraction (XRD).** Prior to XRD analysis, all aqueous dispersions were freeze-dried overnight. Powder XRD analysis was performed using a Bruker D8 ADVANCE X-ray powder diffractometer (Cu-Kα radiation) equipped with a motorized divergence slit for Bragg-Brentano geometry and a high-resolution energy-dispersive Lynxeye XE detector. The mean degree of crystallinity, *D*_c_, for the PCL_24_-TTC precursor and several PCL_24_-PDMAC_x_ diblock copolymers was calculated using diffrac.eva v6.1 software.

**Differential Scanning Calorimetry (DSC).** Measurements were performed using a TA DSC25 Discovery series instrument operating from –90 °C to 200 °C at a heating/cooling rate of 10 °C min^–1^ using aluminum Tzero pans and standard lids. Instrument calibration was performed using an indium standard. All DSC analyses involved three heating/cooling cycles.

**Refractometry.** Absolute refractometry measurements were performed with a Bellingham and Stanley (Royal Tunbridge Wells, UK) handheld OPTI refractometer (Na lamp radiation; λ = 589 nm). Either DMAC or PCL_24_-TTC was heated up to 80 ^o^C, equilibrated for 5 min, and placed on the glass measuring plate.

## **Synthetic Protocols**

**RAFT polymerization of DMAC in the bulk using a PCL_24_-TTC precursor at 80 ^o^C with subsequent dilution with water at an intermediate DMAC conversion**

A 14 mL glass vial was charged with PCL_24_-TTC (0.20 g, 0.065 mmol), DMAC (0.64 g, 6.47 mmol, target DP = 100), ACVA initiator (3.6 mg, 0.013 mmol, [TTC]/[ACVA] molar ratio = 5.0) and a magnetic stirrer bar and sealed with a rubber septum. This vial was placed in an ice bath and deoxygenated with a stream of dry N_2_ gas for 30 min. The vial was allowed to warm up to 20 °C for 10 min before being immersed in an oil bath set at 80 °C. Using a degassed needle and syringe, 0.10 mL aliquots were extracted at 1 min intervals. After 5 min, deoxygenated deionized water (8.46 mL, preheated to 80 °C, targeting 4.5% w/w solids) was added using a degassed syringe/needle. The reaction mixture was periodically sampled throughout using a degassed needle and syringe. The DMAC polymerization was allowed to proceed for 50 min prior to quenching by exposing the reaction mixture to air while cooling to 20 °C.

**UV-initiated RAFT polymerization of DMAC in the bulk using PCL_24_-TTC at 10-30 °C with subsequent dilution with water at an intermediate DMAC conversion**

A 14 mL glass vial was charged with PCL_24_-TTC (0.20 g, 0.065 mmol), DMAC (0.51 g, 5.18 mmol, target DP = 80), AIBN initiator (10.6 mg, 0.065 mmol, [TTC]/[AIBN] molar ratio = 1.0) and a magnetic stirrer bar and sealed with a rubber septum. This vial was placed in an ice bath and its contents were deoxygenated with a stream of dry N_2_ gas for 30 min. The vial was allowed to warm up to 20 °C for 10 min before being placed in a jacketed glass vessel attached to a circulating water bath set at 15 °C. A thermometer was used to confirm that the inside of the vessel was at the same temperature. The DMAC polymerization was initiated by exposing the vessel to UV light (λ = 365 nm) using a Analytik Jena UVP 3UV Lamp (Jena, Germany). After 3.5 h, deoxygenated deionized water (1.69 mL, precooled to 15 °C, targeting 30% w/w solids) was added using a degassed syringe/needle. The reaction vial was then removed from the reactor vessel and subjected to vortex mixing for 5 min to ensure a homogeneous solution, then replaced in the jacketed vessel. At this time point, the reaction mixture was sampled and ^1^H NMR spectroscopy analysis indicated an instantaneous DMAC conversion of 26% (instantaneous PDMAC DP = 21). Subsequently, the reaction mixture was periodically sampled using a degassed needle and syringe. The DMAC polymerization was allowed to proceed for 24 h prior to quenching by exposing the reaction mixture to air. For analogous syntheses in which the target PDMAC DP and reaction temperature were varied, the reagent quantities and volume of added water were adjusted accordingly, as summarized in **Table S1**.

Table S1. Summary of reagent quantities used for the UV-initiated polymerization of DMAC at 10-30 °C using a PCL_24_-TTC precursor, initially in the bulk with subsequent dilution to 30% w/w solids after 3.5 h by addition of deoxygenated deionized water. The instantaneous DMAC conversion on addition of water was determined via ^1^H NMR spectroscopy in each case.

| **Entry Number** | **Target copolymer composition** | **Mass of PCL_24_-TTC**  **grams** | **Mass of DMAC**  **grams** | **Mass of AIBN initiator**  **mg** | **Volume of added water**  **mL** | **Dilution time**  **h** | **Reaction Temperature**  **°C** | **Intermediate DMAC conversion**  **%** | **Final DMAC conversion**  **%** |
| --- | --- | --- | --- | --- | --- | --- | --- | --- | --- |
| 1 | PCL_24_-PDMAC_60_ | 0.20  (0.065 mmol) | 0.39  (3.88 mmol) | 10.6  (6.5 µmol) | 1.39 | 3.5 | 15 | 56 | 92 |
| 2 | PCL_24_-PDMAC_80_ | 0.20  (0.065 mmol) | 0.51  (5.18 mmol) | 10.6  (6.5 µmol) | 1.69 | 3.5 | 15 | 26 | 93 |
| 3 | PCL_24_-PDMAC_100_ | 0.20  (0.065 mmol) | 0.64  (6.47 mmol) | 10.6  (6.5 µmol) | 1.99 | 3.5 | 15 | 33 | 93 |
| 4 | PCL_24_-PDMAC_80_ | 0.20  (0.065 mmol) | 0.51  (5.18 mmol) | 10.6  (6.5 µmol) | 1.69 | 3.5 | 10 | 22 | 80 |
| 5 | PCL_24_-PDMAC_80_ | 0.20  (0.065 mmol) | 0.51  (5.18 mmol) | 10.6  (6.5 µmol) | 1.69 | 1.5 | 30 | 35 | >99 |

# **Additional Characterization Data**


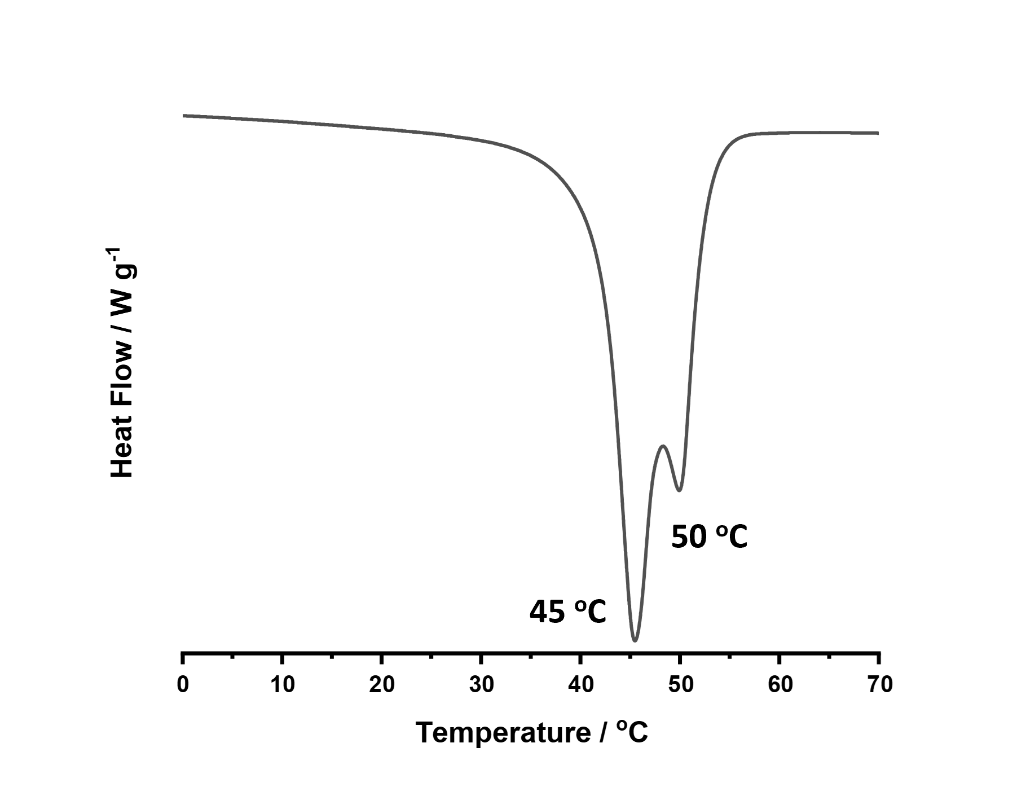


**Figure S1**. DSC curve recorded for the PCL_24_-TTC precursor (*T*_m_ = 45 °C) at a heating rate of 10 °C min^-1^.


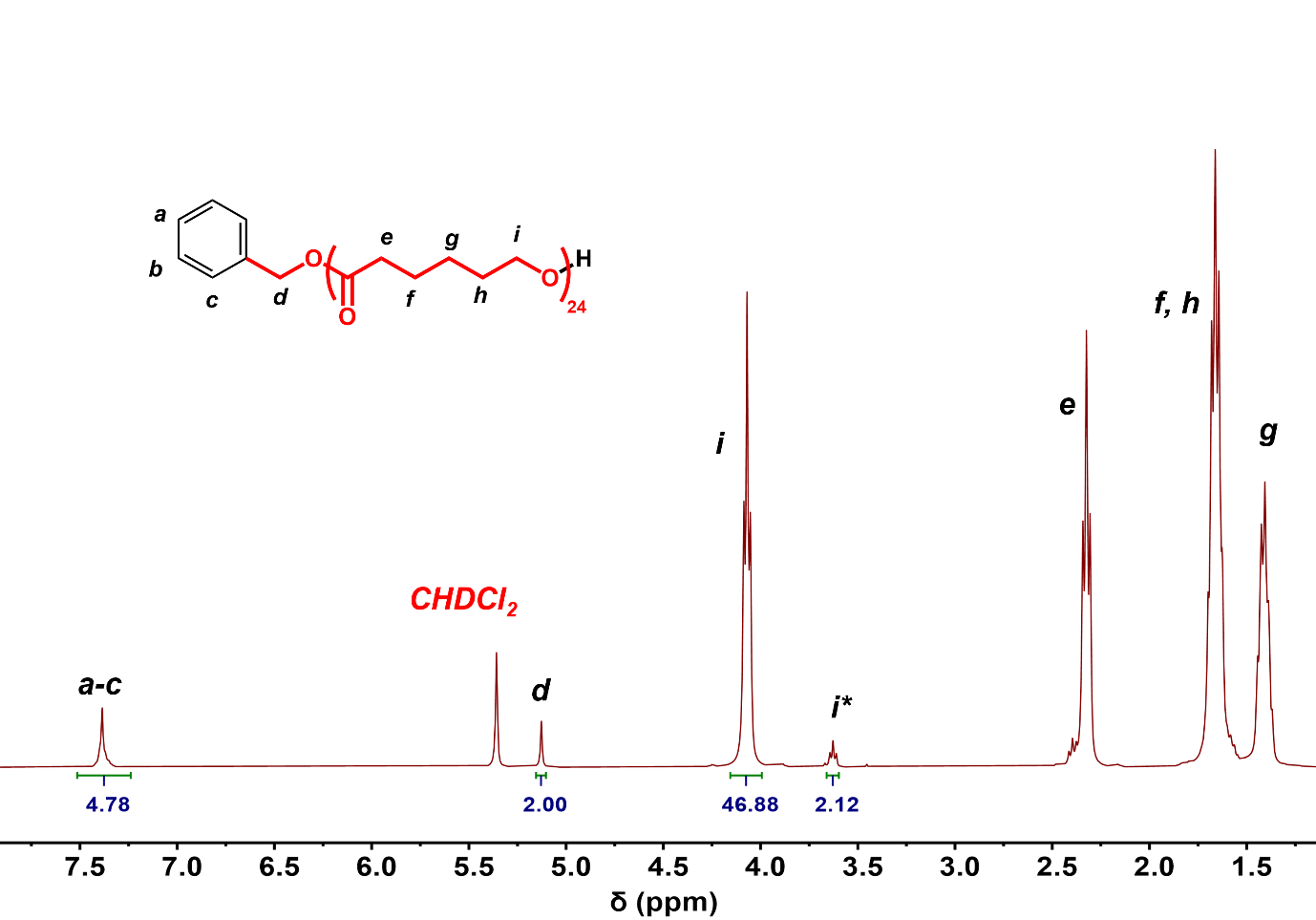


**Figure S2**. Assigned ^1^H NMR spectrum (CD_2_Cl_2_) recorded for the PCL_24_-OH precursor, which was prepared via TBD-catalyzed anionic ring-opening polymerization of ε-caprolactone using benzyl alcohol as an initiator.


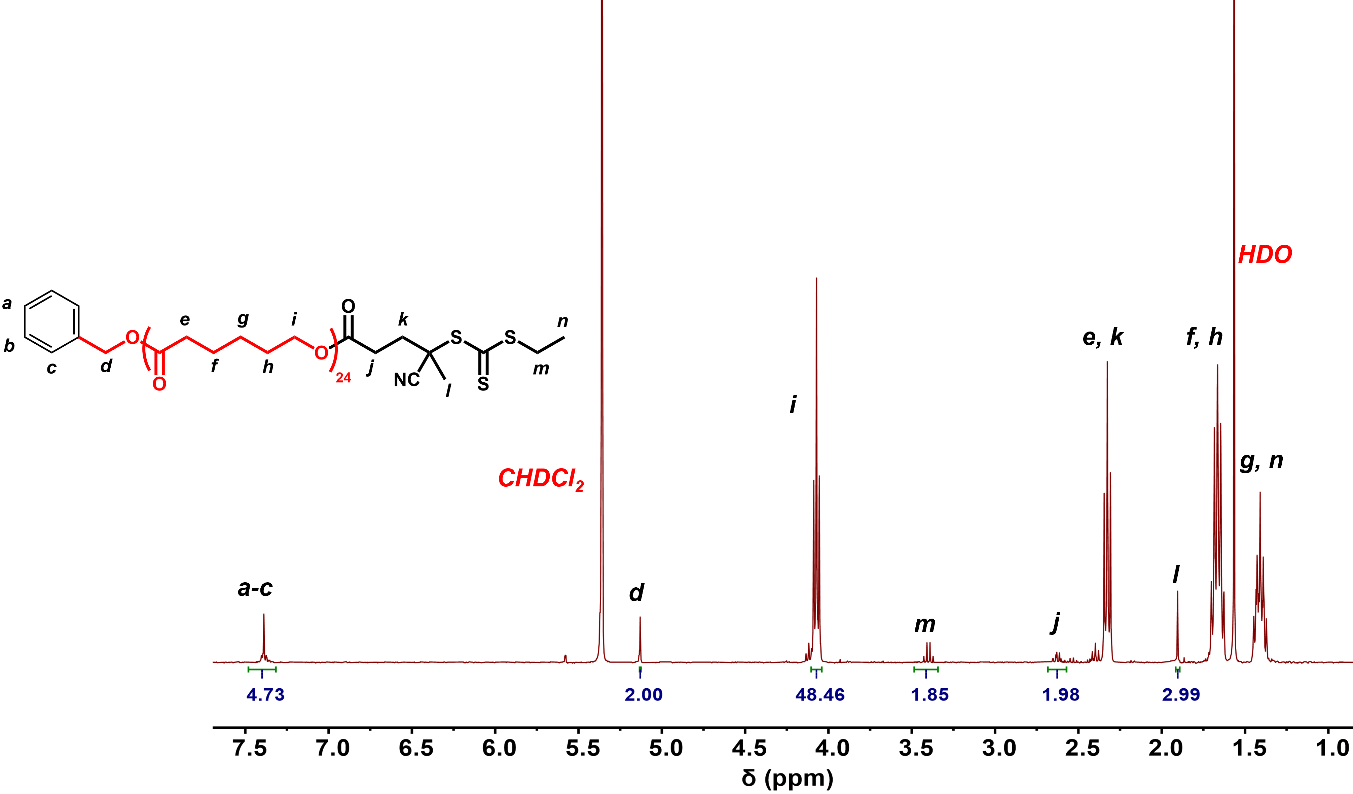


**Figure S3**. Assigned ^1^H NMR spectrum (CD_2_Cl_2_) recorded for a PCL_24_-TTC precursor, which was prepared by DCC/DMAP-catalyzed esterification of a hydroxy-capped PCL_24_ precursor using an excess of a carboxylic acid-functionalized RAFT agent (CEPA).


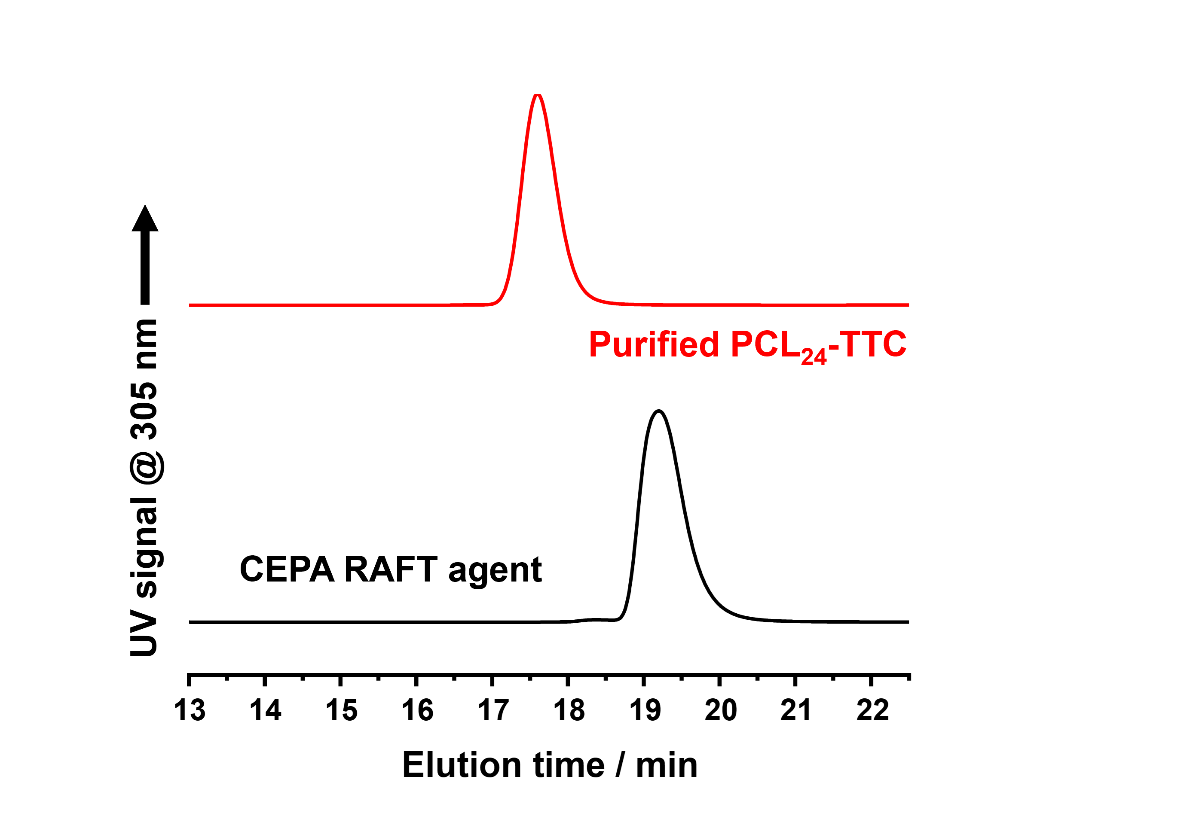


**Figure S4**. DMF UV GPC curves (λ = 305 nm) recorded for PCL_24_-TTC (red curve) and the RAFT agent CEPA (black curve). For GPC analysis of the RAFT agent, the DMF eluent contained 1.0% glacial acetic acid but no LiBr. For GPC analysis of the PCL_24_-TTC, the DMF eluent contained 10 mM LiBr but no glacial acetic acid.


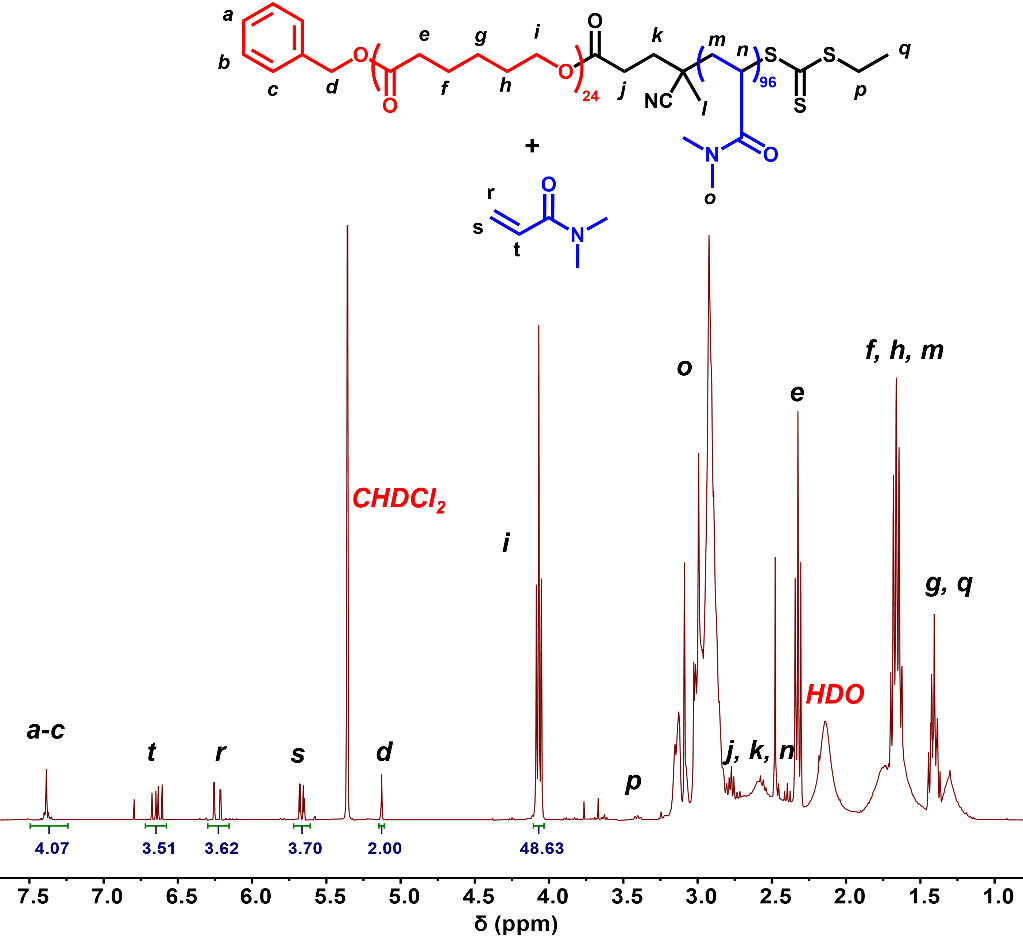


**Figure S5**. Assigned ^1^H NMR spectrum (CD_2_Cl_2_) recorded for the PCL_24_-PDMAC­_96_ diblock copolymer obtained at the end of the TR-SAXS experiment.


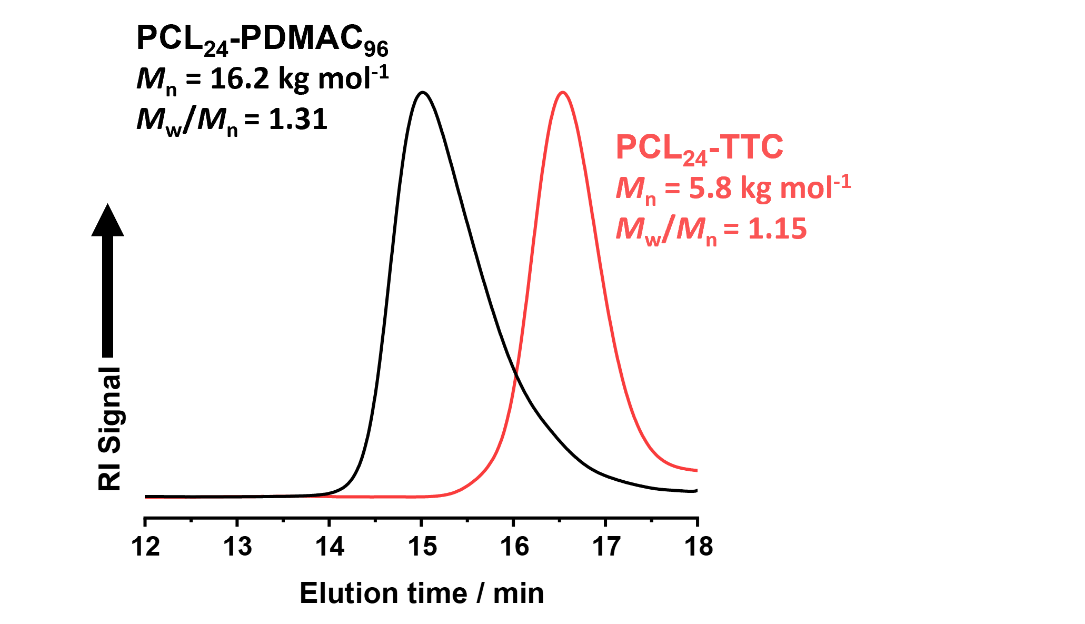


**Figure S6**. DMF GPC curves (refractive index detector) recorded for the PCL_24_-TTC precursor (red curve) and the corresponding PCL_24_-PDMAC_96_ diblock copolymer (black curve) obtained at the end of the TR-SAXS experiment.


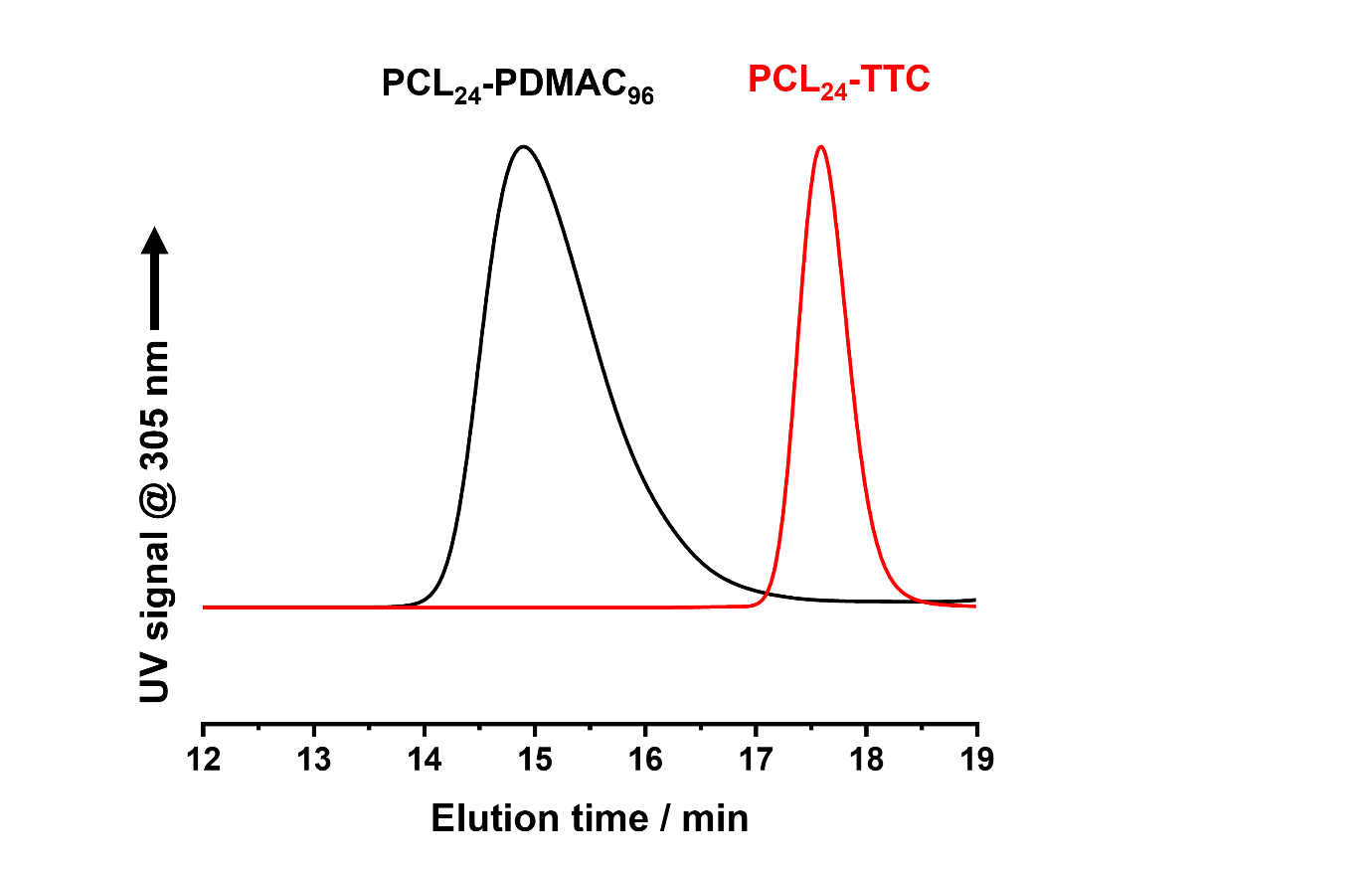


**Figure S7**. DMF UV GPC curves (λ = 305 nm) recorded for the PCL_24_-TTC precursor (red curve) and the corresponding PCL_24_-PDMAC_96_ diblock copolymer (black curve) obtained at the end of the TR-SAXS experiment.


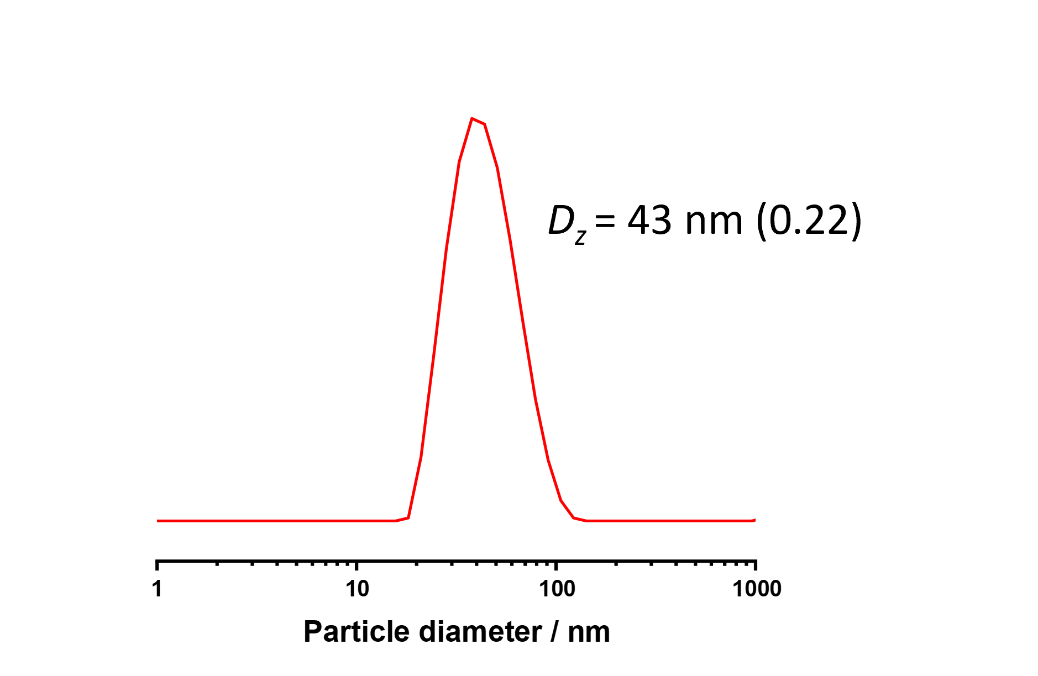


**Figure S8**. DLS particle size distribution recorded for a 0.1% w/w aqueous dispersion of the PCL_24_-PDMAC­_96_ diblock copolymer nanoparticles obtained at the end of the TR-SAXS experiment. The hydrodynamic z-average diameter, *D*_z_, = 43 nm and the DLS polydispersity index = 0.22.


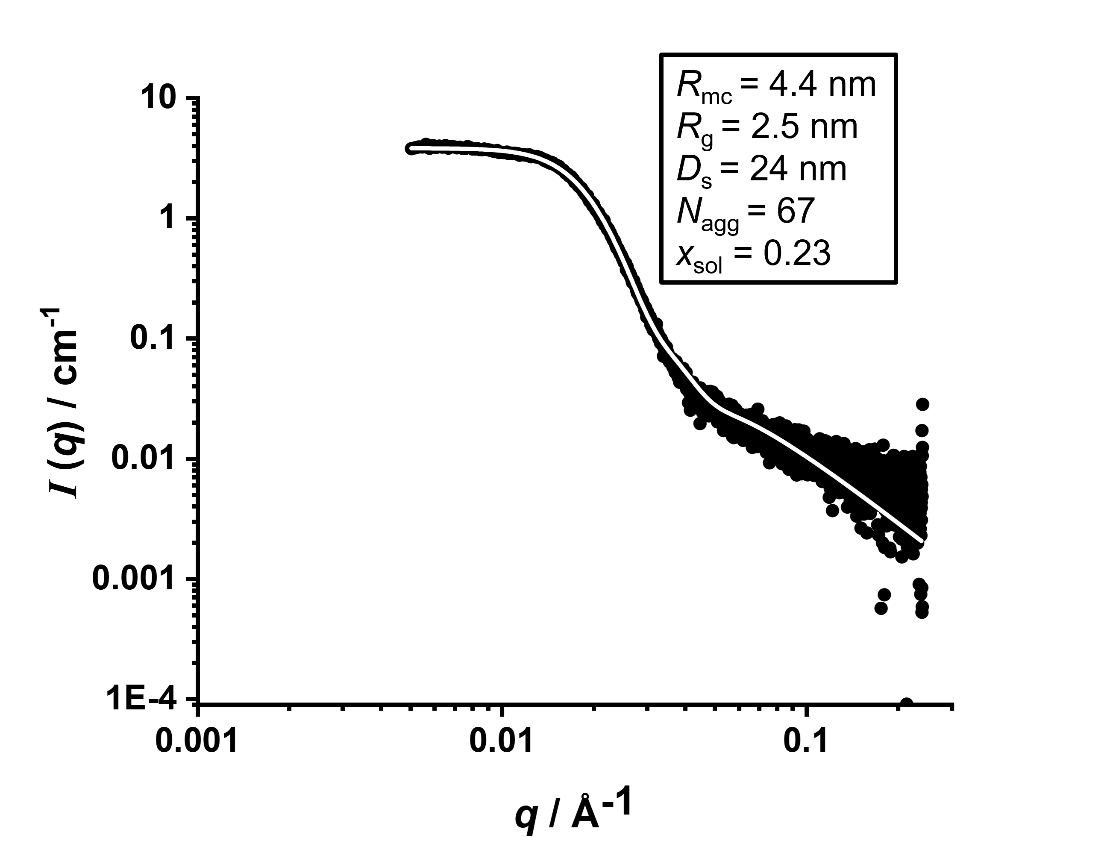


**Figure S9.** SAXS pattern recorded after 50 min (final frame) during TR-SAXS studies of the synthesis of PCL_24_-PDMAC_96_ nanoparticles (black data points) via *reverse sequence* aqueous PISA. The data fit (white curve) was obtained using a well-known spherical micelle model (see **Equation S3**),^7^ where *R*_mc_ is the mode value of the micelle core radius, *R*_g_ is the radius of gyration of the corona block, *N*_agg_ is the mode value of the aggregation number and *D*_s_ is the mode value of the total particle diameter (*D*_s_ = 2*R*_mc_ + 2*s* = 2*R*_mc_ + 6*R_g_*), where *s* describes the width of the micelle corona profile, see **Figure S10**.


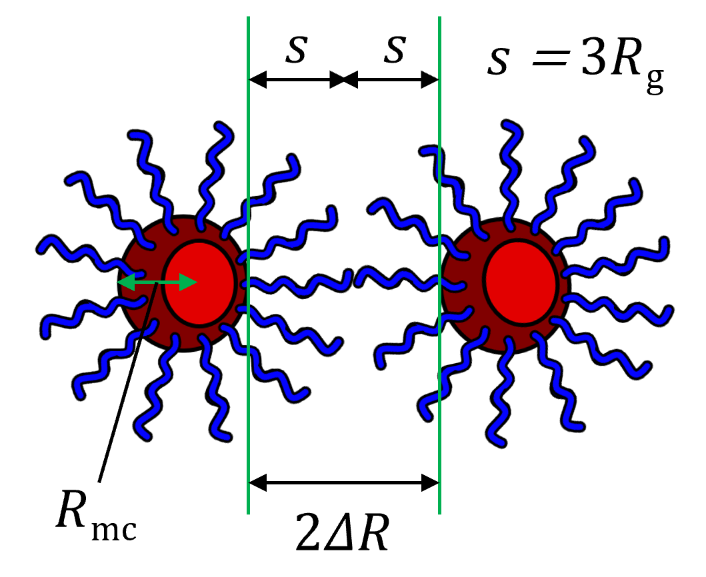


**Figure S10.** Schematic cartoon illustrating the four fitting parameters used in the spherical micelle model,^7^ where *s* is the width of the micelle corona profile, *R*_mc_ is the nanoparticle core radius, *R*_g_ is the radius of gyration of the corona block and 2$\Delta R$ is the shortest distance between interfaces of the neighbouring micelle cores.

**
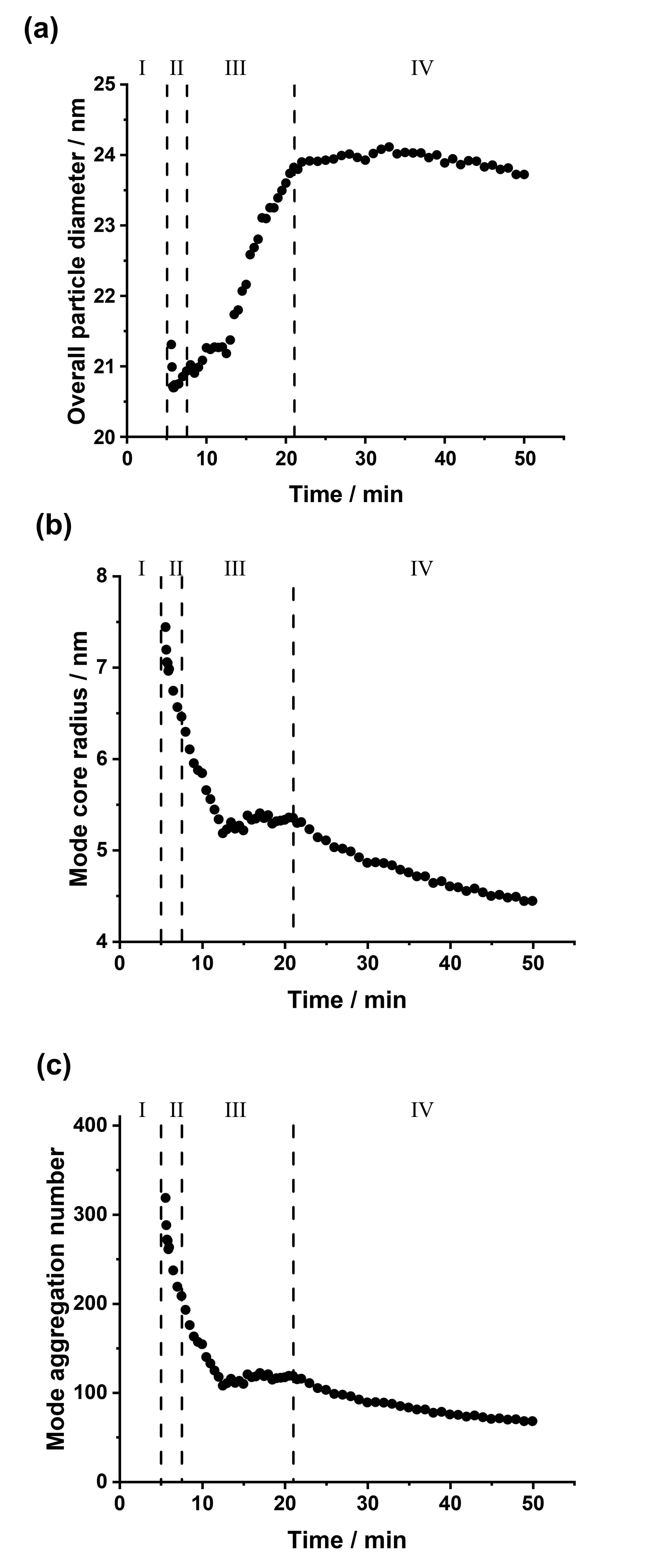
**

**Figure S11**. (a) Evolution of the overall nanoparticle diameter, *D*_s_, (b) mode core radius, *R*_mc_, and (c) mode aggregation number during the synthesis of PCL_24_-PDMAC_96_ spherical nanoparticles by *reverse sequence* aqueous PISA, as judged by TR-SAXS studies.

**
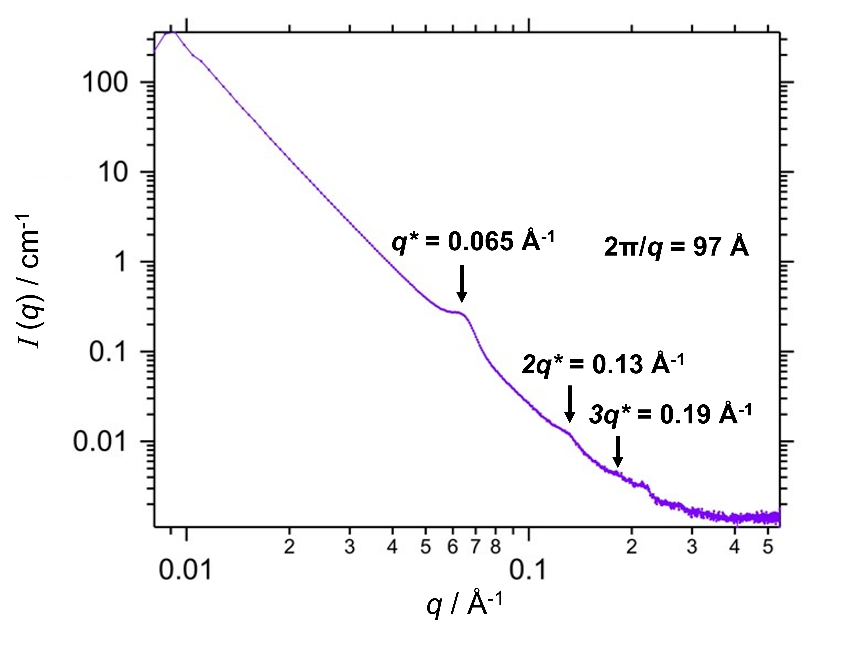
**

**Figure S12**. SAXS pattern recorded for the solid PCL_24_-TTC precursor used to prepare the PCL_24_-PDMAC_96_ nanoparticles reported in this study. The structure peak at *q** ~ 0.065 Å^-1^ with the associated higher order peaks (2*q** ~ 0.13 Å^-1^ and 3*q** ~ 0.19 Å^-1^) correspond to a lamellar period, *d*, of 97 Å or 9.7 nm (as calculated using the well-known approximation, *d* = 2π/*q**).^13^


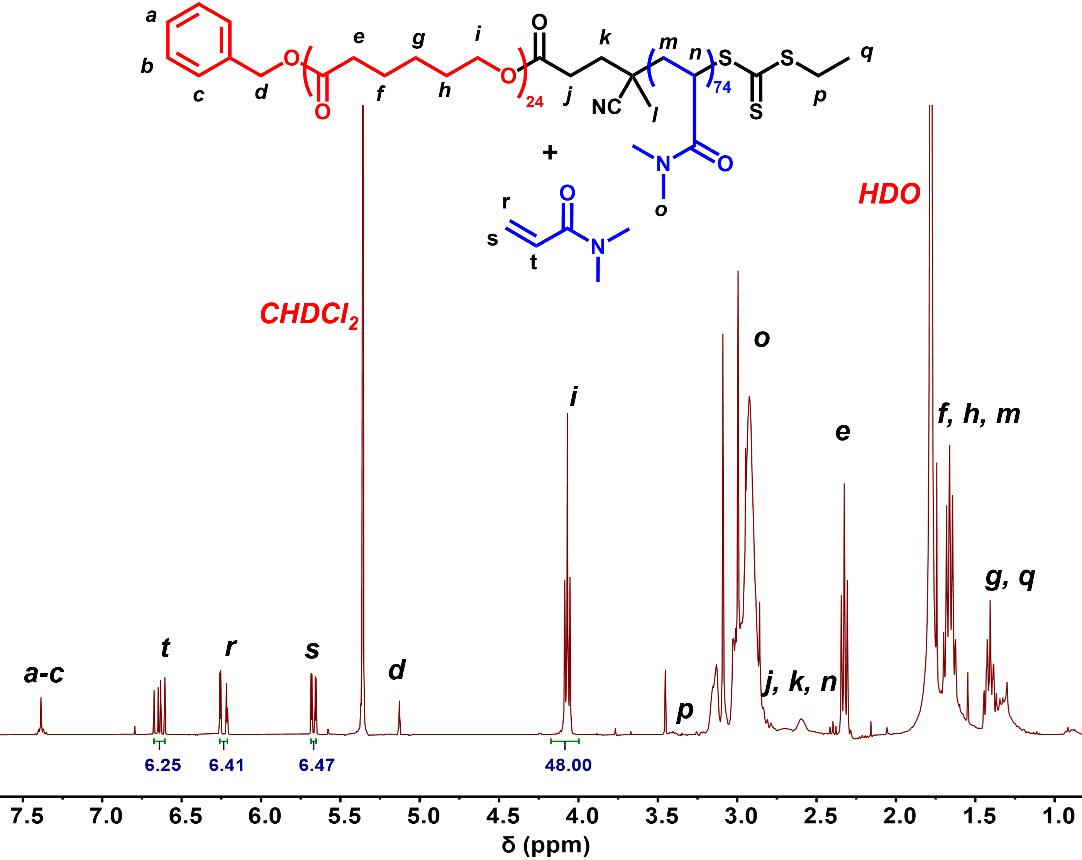


**Figure S13**. Assigned ^1^H NMR spectrum (CD_2_Cl_2_) recorded for the PCL_24_-PDMAC­_74_ diblock copolymer prepared by UV-initiated polymerization of DMAC at 15 °C using the PCL_24_-TTC precursor and AIBN initiator.


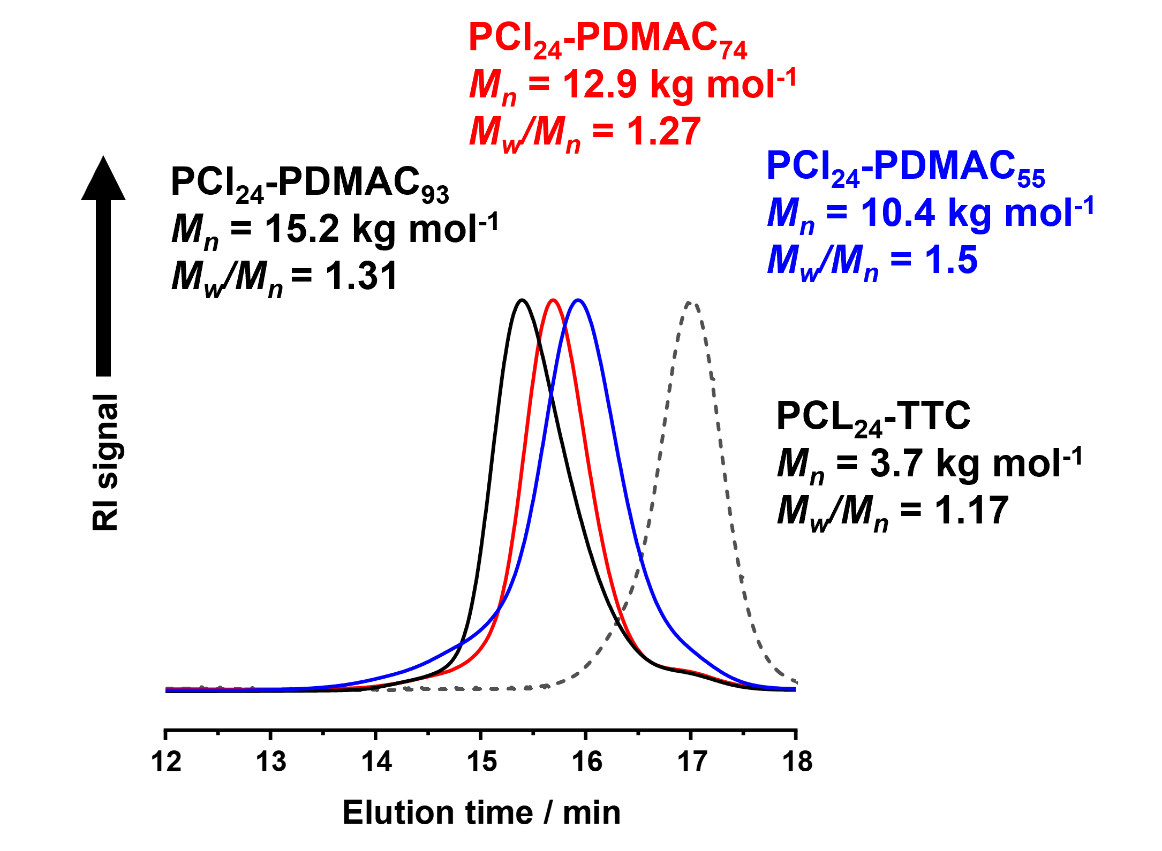


**Figure S14**. DMF GPC curves (refractive index detector) recorded for the PCL_24_-TTC precursor (black dashed trace) and the PCL_24_-PDMAC_x_ diblock copolymer prepared by UV-initiated polymerization of DMAC at 15 °C using this PCL_24_-TTC precursor plus AIBN initiator, when targeting a PDMAC DP of 60 (blue trace), 80 (red trace), or 100 (black trace).

**
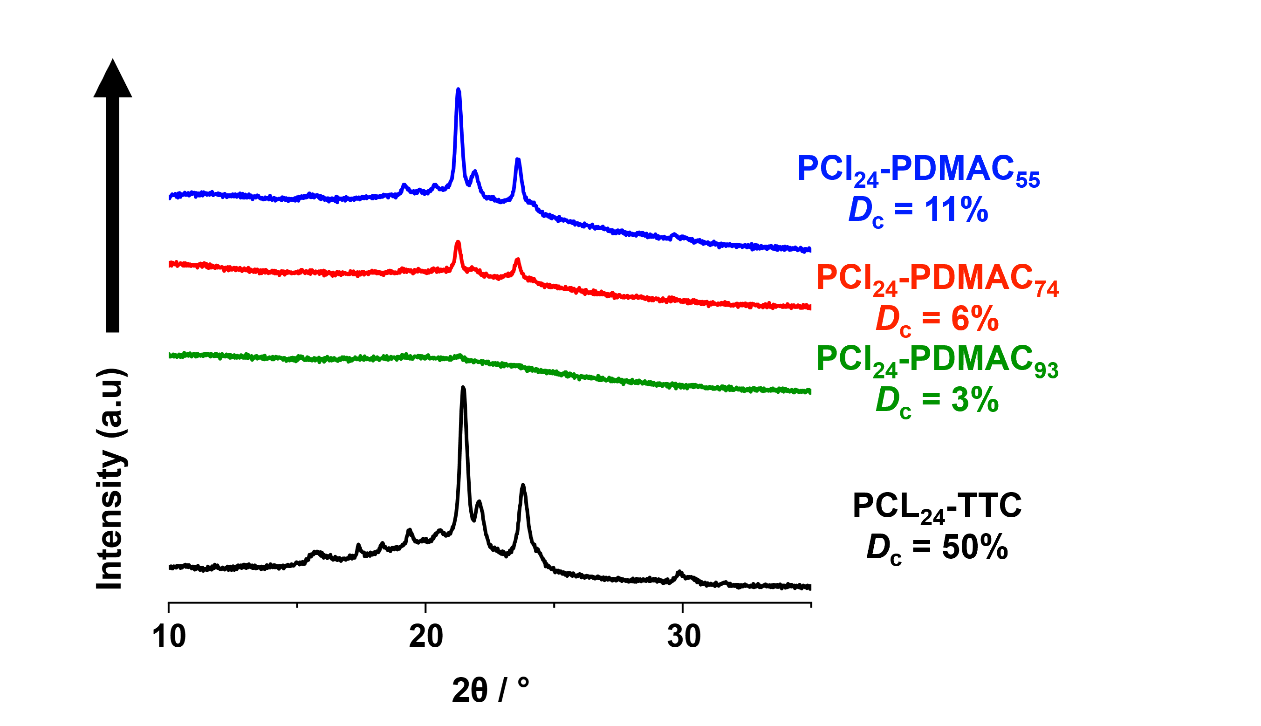
**

**Figure S15.** XRD patterns recorded for PCL_24_-PDMAC_55_ (blue curve), PCL_24_-PDMAC_74_ (red curve) and PCL_24_-PDMAC_93_ (green curve) diblock copolymers (in bulk) prepared by UV-initiated polymerization of DMAC at 15 °C.


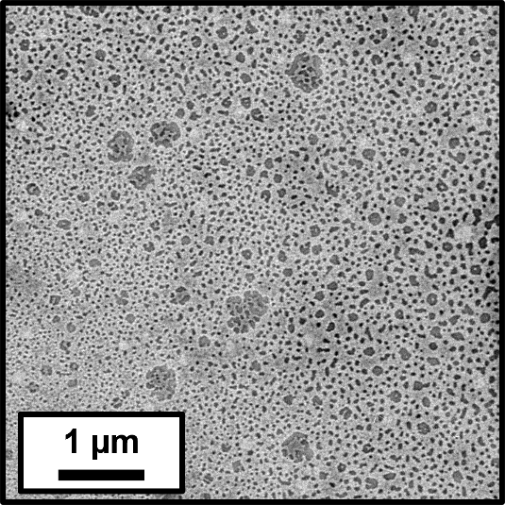


**Figure S16.** Representative TEM image recorded for a dilute aqueous dispersion of PCL_24_-PDMAC_93_ nanoparticles prepared by UV-initiated polymerization of DMAC at 15 °C (see entry 3 in Table S1).

**
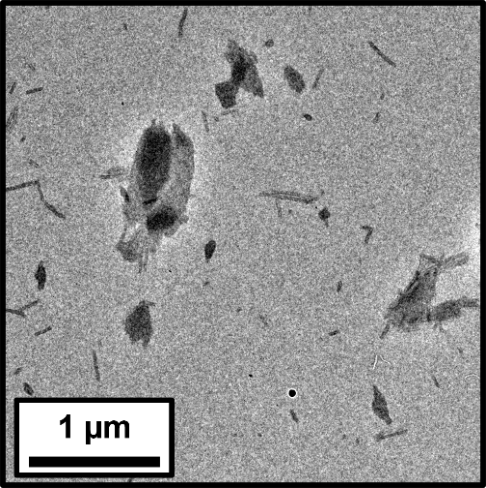
**

**Figure S17.** Representative TEM image recorded for a dilute aqueous dispersion of aggregated PCL_24_-PDMAC_55_ rod-like nanoparticles prepared by UV-initiated polymerization of DMAC at 15 °C (see entry 1 in Table S1).


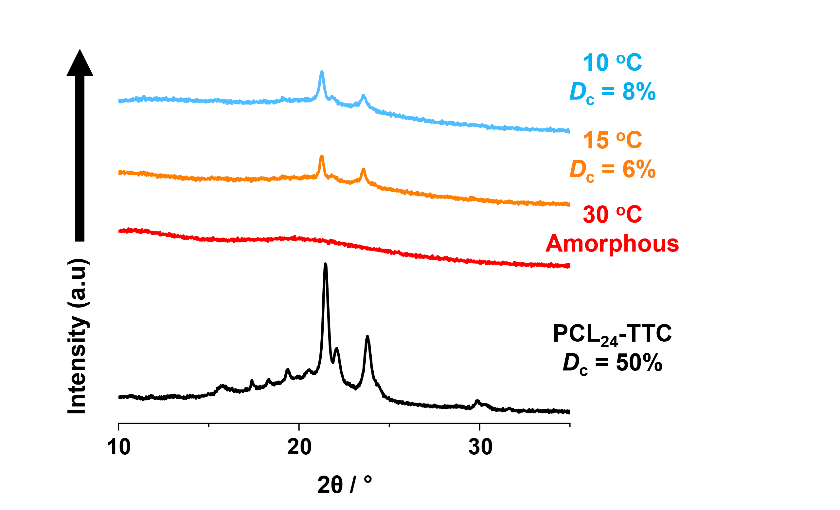


**Figure S18.** XRD patterns recorded for three PCL_24_-PDMAC_80_ diblock copolymers (in bulk) prepared by UV-initiated polymerization at 10 °C (blue curve), 15 ^o^C (orange curve) or 30 °C (red trace), plus the corresponding PCL_24_-TTC precursor (black curve).


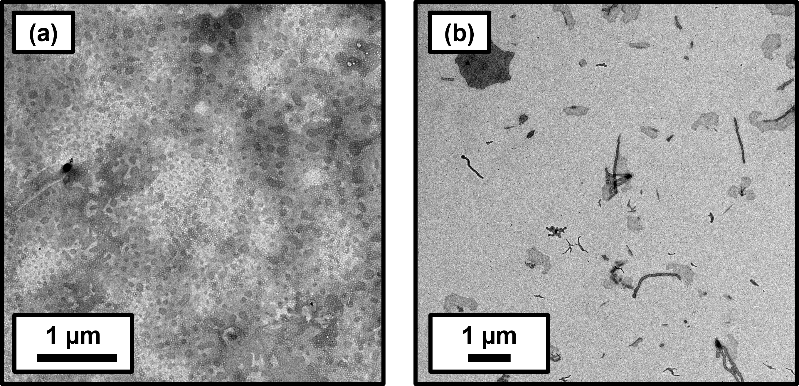


**Figure S19.** Representative TEM images recorded for a dilute aqueous dispersion of PCL_24_-PDMAC_x_ diblock copolymer nanoparticles (target DP, x = 80) prepared by UV-initiated polymerization at (a) 30 ^o^C and (b) 10 °C (see entries 5 and 4 in Table S1, respectively).


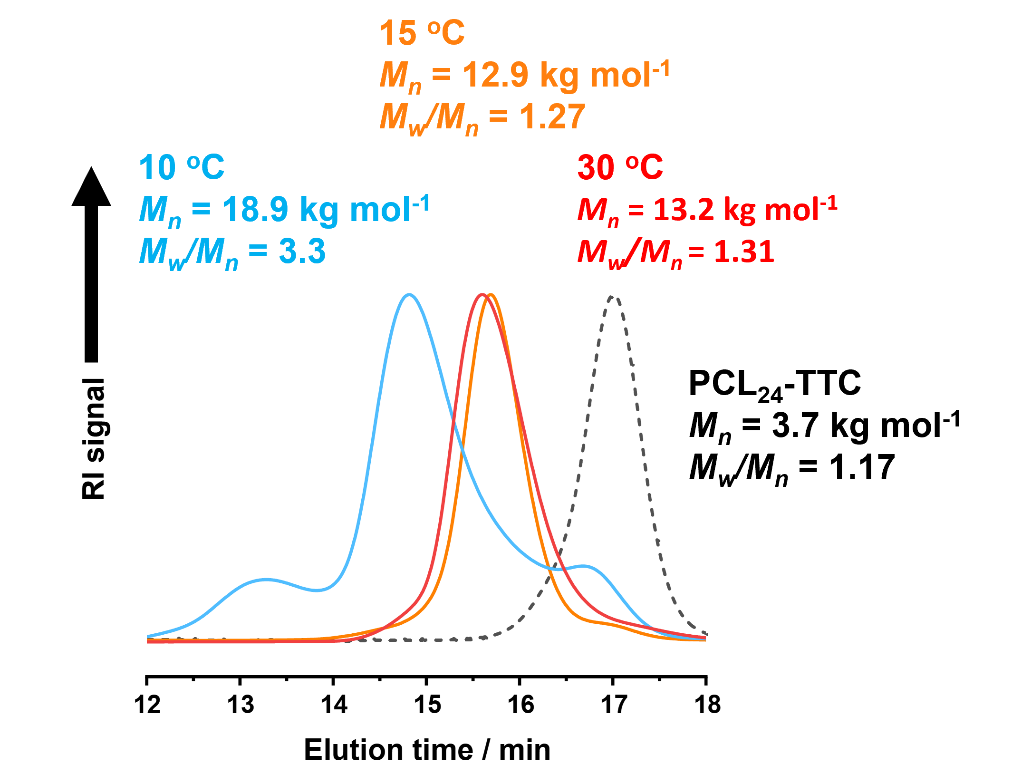


**Figure S20.** DMF RI GPC curves (refractive index detector) recorded for three PCL_24_-PDMAC_x_ (target DP x = 80) diblock copolymers synthesized at 10 °C (blue curve), 15 °C (orange curve) and 30 °C (red curve). The PCL_24_-TTC precursor (black curve, refractive index detector) is also shown as a reference.

# **References**

[1] M. A. H. Farmer, O. M. Musa, S. P. Armes, *Angew. Chem. Int. Ed.* **2023**, *62*, e202309526.

[2] M. C. Arno, M. Inam, Z. Coe, G. Cambridge, L. J. Macdougall, R. Keogh, A. P. Dove, R. K. O’Reilly, *J. Am. Chem. Soc.* **2017**, *139*, 16980–16985.

[3] T. Narayanan, M. Sztucki, T. Zinn, J. Kieffer, A. Homs-Puron, J. Gorini, P. Van Vaerenbergh, P. Boesecke, *J. Appl. Crystallogr.* **2022**, *55*, 98–111.

[4] J. Filik, A. W. Ashton, P. C. Y. Chang, P. A. Chater, S. J. Day, M. Drakopoulos, M. W. Gerring, M. L. Hart, O. V. Magdysyuk, S. Michalik, A. Smith, C. C. Tang, N. J. Terrill, M. T. Wharmby, H. Wilhelm, *J. Appl. Crystallogr.* **2017**, *50*, 959–966.

[5] J. Ilavsky, P. R. Jemian, *J. Appl. Crystallogr.* **2009**, *42*, 347–353.

[6] A. J. Smith, S. G. Alcock, L. S. Davidson, J. H. Emmins, J. C. Hiller Bardsley, P. Holloway, M. Malfois, A. R. Marshall, C. L. Pizzey, S. E. Rogers, O. Shebanova, T. Snow, J. P. Sutter, E. P. Williams, N. J. Terrill, *J. Synchrotron Radiat.* **2021**, *28*, 939–947.

[7] J. S. Pedersen, *Adv. Colloid Interface Sci.* **1997**, *70*, 171–210.

[8] J. S. Pedersen, M. C. Gerstenberg, *Colloids Surfaces A Physicochem. Eng. Asp.* **2003**, *213*, 175–187.

[9] S. J. Byard, C. T. O’Brien, M. J. Derry, M. Williams, O. O. Mykhaylyk, A. Blanazs, S. P. Armes, *Chem. Sci.* **2020**, *11*, 396–402.

[10] S. Förster, C. Burger, *Macromolecules* **1998**, *31*, 879–891.

[11] D. J. Kinning, E. L. Thomas, *Macromolecules* **1984**, *17*, 1712–1718.

[12] J. S. Pedersen, *J. Chem. Phys.* **2001**, *114*, 2839–2846.

[13] O. J. Deane, J. Jennings, T. J. Neal, O. M. Musa, A. Fernyhough, S. P. Armes, *Chem. Mater.* **2021**, *33*, 7767–7779.
